# Species-specific alternative splicing leads to unique expression of *sno-lncRNAs*

**DOI:** 10.1186/1471-2164-15-287

**Published:** 2014-04-16

**Authors:** Xiao-Ou Zhang, Qing-Fei Yin, Hai-Bin Wang, Yang Zhang, Tian Chen, Ping Zheng, Xuhua Lu, Ling-Ling Chen, Li Yang

**Affiliations:** 1 Key Laboratory of Computational Biology, CAS-MPG Partner Institute for Computational Biology, Shanghai Institutes for Biological Sciences, Chinese Academy of Sciences, Shanghai 200031, China; 2 State Key Laboratory of Molecular Biology, Institute of Biochemistry and Cell Biology, Shanghai Institutes for Biological Sciences, Chinese Academy of Sciences, Shanghai 200031, China; 3 Department of Orthopedic Surgery, Changzheng Hospital, Second Military Medical University, Shanghai 200003, China; 4 State Key Laboratory of Genetic Resources and Evolution, Kunming Institute of Zoology, Chinese Academy of Sciences, Kunming, Yunnan 650223, China

**Keywords:** lncRNA, *sno-lncRNA*, Alternative splicing, Species-specific, PWS

## Abstract

**Background:**

Intron-derived long noncoding RNAs with snoRNA ends (*sno-lncRNAs*) are highly expressed from the imprinted Prader-Willi syndrome (PWS) region on human chromosome 15. However, *sno-lncRNAs* from other regions of the human genome or from other genomes have not yet been documented.

**Results:**

By exploring non-polyadenylated transcriptomes from human, rhesus and mouse, we have systematically annotated *sno-lncRNAs* expressed in all three species. In total, using available data from a limited set of cell lines, 19 *sno-lncRNAs* have been identified with tissue- and species-specific expression patterns. Although primary sequence analysis revealed that snoRNAs themselves are conserved from human to mouse, *sno-lncRNAs* are not. PWS region *sno-lncRNAs* are highly expressed in human and rhesus monkey, but are undetectable in mouse. Importantly, the absence of PWS region *sno-lncRNAs* in mouse suggested a possible reason why current mouse models fail to fully recapitulate pathological features of human PWS. In addition, a *RPL13A* region *sno-lncRNA* was specifically revealed in mouse embryonic stem cells, and its snoRNA ends were reported to influence lipid metabolism. Interestingly, the *RPL13A* region *sno-lncRNA* is barely detectable in human. We further demonstrated that the formation of *sno-lncRNAs* is often associated with alternative splicing of exons within their parent genes, and species-specific alternative splicing leads to unique expression pattern of *sno-lncRNAs* in different animals.

**Conclusions:**

Comparative transcriptomes of non-polyadenylated RNAs among human, rhesus and mouse revealed that the expression of *sno-lncRNAs* is species-specific and that their processing is closely linked to alternative splicing of their parent genes. This study thus further demonstrates a complex regulatory network of coding and noncoding parts of the mammalian genome.

## Background

Although only about 2% of the human genome encodes protein sequences [1,2], recent advances in genomewide analyses have revealed that the majority of the human genome is transcribed [3,4], largely from noncoding segments that used to be considered as “junk sequences” or “dark matter” [5,6]. Besides well-characterized housekeeping noncoding RNAs (such as tRNA, rRNA, snRNA and snoRNA) and small regulatory ncRNAs [7,8], the transcriptome has become even more complex with pervasively transcribed long noncoding RNAs (lncRNAs, at least 200 nt long) [4,9,10]. Using systematic and integrative strategies and by considering multiple biological features, thousands of lncRNAs were identified from intergenic regions (long intergenic noncoding RNAs, lincRNAs) in mouse [11], zebrafish [12] and human [13] genomes. Importantly, the strategy of lincRNA discovery has served as a road map for the systematic annotation of other lncRNAs.

In addition to intergenic regions, introns account for over 20% of noncoding sequences in the human genome and provide yet another source to generate lncRNAs. By removal of redundant rRNAs and poly(A)+ RNAs, a relatively pure population of non-polyadenylated and non-ribosomal (poly(A)-/ribo-) RNAs was obtained and subjected to high-throughput deep sequencing [14]. This type of poly(A)- RNA-seq of the human cell transcriptomes surprisingly revealed previously-ignored RNA signals in exons and introns [14-16]. Interestingly, nuclear fractionation also indicated the presence of stable transcripts from intronic sequences in *X. tropicali*s [17]. What mechanism(s) can protect these excised introns from rapid degradation after splicing? Further analyses revealed a class of intron-derived lncRNAs that depend on the snoRNA machinery at both ends for their processing (*sno-lncRNAs*) [15]. This finding shed new light on lncRNA characterization from “junk” intronic sequences.

Strikingly, five *sno-lncRNAs* derived from introns of the Prader-Willi syndrome (PWS) region (15q11-q13) were highly expressed in human embryonic stem cells and strongly associated with Fox family splicing regulators to alter patterns of splicing [15]. The PWS 15q11-q13 region is imprinted, leading to the expression of the SNURF-SNRPN gene and downstream noncoding region from the paternal chromosome. All paternal transcripts downstream of the SNRPN gene are noncoding and have been considered primarily as precursors for small RNAs, including the SNORD116 cluster of 29 similar snoRNAs [18,19]. Importantly, SNORD116 deficiency has been recognized as the primary cause of PWS in recent disease model [20-22]. Although the function of SNORD116s remained elusive, the recently identification of PWS region *sno-lncRNAs* and their association with Fox family splicing regulators offers a functional connection of *sno-lncRNAs* in the molecular pathogenesis of PWS [15]. However, it was not clear how many other *sno-lncRNAs* may exist in the genome. Given that the vast majority of snoRNAs are encoded in introns of protein-coding genes [23], it was of interest to annotate *sno-lncRNAs* in a genomewide manner. Moreover, signals of poly(A)- transcripts from intronic regions have been widely detected in a variety of cultured cells [10], which has provided a rich data source to explore *sno-lncRNAs* from different cell lines.

Here, we applied computational pipelines to identify *sno-lncRNAs* genome-widely from poly(A)-/ribo- transcriptomes of human, rhesus and mouse. In total, 19 *sno-lncRNAs* have been identified with tissue- and species-specific expression patterns from available species/cell lines. PWS region *sno-lncRNAs* are highly expressed in human, somewhat in rhesus, and none in mouse. In contrast, a *RPL13A* region *sno-lncRNA* is highly expressed in mouse, but almost absent in human. We further demonstrated that the formation of *sno-lncRNAs* often requires alternative splicing, indicating a complex regulatory network of coding and noncoding parts of the genome.

## Results and discussion

### Genomewide identification of *sno-lncRNAs* across species

**Figure 1:**
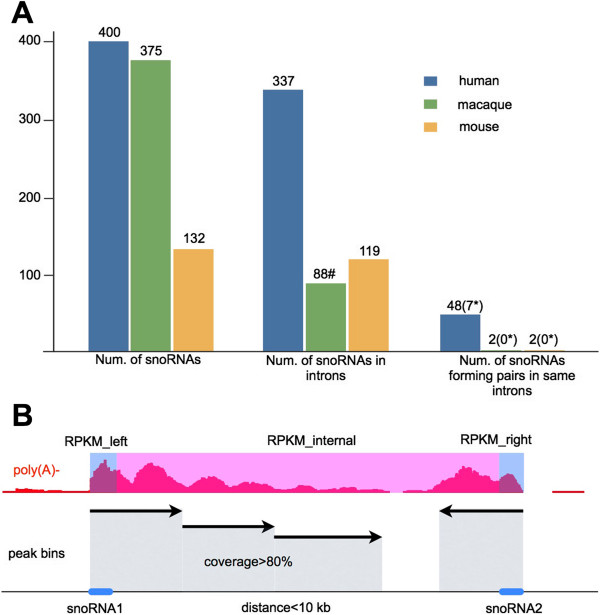
**Genomewide prediction of *sno-lncRNAs*. (A)** Genomewide prediction of *sno-lncRNAs* with annotated snoRNAs. The numbers of annotated snoRNAs, snoRNAs in introns or snoRNAs forming pairs in any single intron were compared across human (blue), rhesus (green) and mouse (yellow). Rhesus snoRNAs were transposed from human and mouse annotations. Note that only a few of snoRNAs could form pairs in any single intron, and even fewer *sno-lncRNAs* (highlighted by asterisk) could be detected with poly(A)-/ribo- RNA-seq signals. **(B)** Genomewide prediction of *sno-lncRNAs* with an intron-annotation-independent algorithm. For each two adjacent snoRNAs (blue bars) within 10 kb, RPKM of snoRNAs (blue regions) and internal region (pink regions) in poly(A)-/ribo- RNA-seq datasets were evaluated with a set of criteria to identify novel *sno-lncRNAs* (Methods). In total, 19 *sno-lncRNAs* were identified in examined species and cell lines (Table 1).

The processing of intron-derived *sno-lncRNAs* depends on the snoRNA machinery at both ends [15]. Five such *sno-lncRNAs* are highly expressed in introns of PWS imprinted region of chr15 and are strongly associated with Fox family splicing regulators to alter patterns of splicing [15]. However, *sno-lncRNAs* from other regions of the human genome or other species were less obvious. Since the vast majority of snoRNA genes are located in introns of protein-coding genes (Figure 1A) [23], and one intron containing two snoRNA genes is a prerequisite for the generation of a *sno-lncRNA*, we first surveyed genomic locations of annotated snoRNAs in different genomes to locate snoRNA pairs in one intron (≥two snoRNAs/intron). In total, 400 annotated snoRNAs were downloaded from snoRNABase [24] for human, and 132 snoRNAs for mouse from RefSeq (http://www.ncbi.nlm.nih.gov/refseq/, downloaded on 2013/3/4), respectively. Since snoRNAs in rhesus are not well annotated, we transposed human and mouse snoRNA annotations over to the rhesus genome to generate 375 putative rhesus snoRNAs (Methods). We then analyzed the genomic locations of these snoRNAs in different species.

Only a small portion of snoRNA pairs could be found from any single RefSeq intron. Importantly, even fewer such introns are expressed in detected cell lines by interrogating poly(A)-/ribo- RNA-seq datasets. For example, in human pluripotent H9 cells, only seven *sno-lncRNAs* could be detected, including six reported *sno-lncRNAs*[15] and a new one derived from the *C17orf76-AS1* region (Table 1), although we identified 48 snoRNAs that form pairs within single introns (Figure 1A). In addition, no putative *sno-lncRNAs* could be detected in rhesus or mouse by analyzing snoRNA pairs in annotated introns.

**Table 1:** **Summary of *sno-lncRNAs *** **identified from human, rhesus and mouse ESCs**

**Species**	**Cell line**	**Location**	**Expression level (RPKM)**	**snoRNA caps**	**Parent gene**	**Validated by NB**	**Notes**
Human	ESC (H9)	chr15:25310171-25313030	142	SNORD116-6, SORD116-7	SNURF-SNRPN	Yes	sno-IncRNA1
		chr15:25324203-25325381	829	SNORD116-13, SNORD116-14	SNURF-SNRPN	Yes	sno-IncRNA2
		chr15:25330530-25331766	2319	SNORD116-18, SNORD116-19	SNURF-SNRPN	Yes	sno-IncRNA3
		chr15:25332807-25334043	1012	SNORD116-20, SNORD116-21	SNURF-SNRPN	Yes	sno-IncRNA4
		chr15:25344644-25346814	113	SNORD116-26, SNORD116-27	SNURF-SNRPN	Yes	sno-IncRNA5
		chr7:45143947-45144641	186	SNORA5A, SNORA5C	TBRG4	Yes	sno-Inc5AC
		chr17:16342822-16343420	106	SNORD49B, SNORD49A	C17orf76-AS1	Yes	Additional file 1
		chr11:93463680-93464265	18	SNORA25, SNORA32	TAF1D	Yes	Additional file 2
	ENCODE Cell Lines	chr1:173833312-173833583	29	SNORD81, SNORD47	GAS5	N/A	N/A
		chr9:136216948-136217382	14	SNORD36B, Snord36A	RPL7A	N/A	N/A
		chr11:93466393-93466763	8	SNORD5, SNORA18*	TAD1D	N/A	N/A
Rhesus	ESC	chr1:204218457-204219170	14	SNORD78, SNORD77	GAS5	N/A	N/A
		chr1:204219672-204220510	18	SNORD75, SNORD74	GAS5	N/A	N/A
		chr7:2838589-2840464	83	SNORD116, SNORD116	SNURF-SNRPN	N/A	human sno-IncRNA3 homologue
		chr7:2850511-2851734	34	SNORD116, SNORD116	SNURF-SNRPN	N/A	human sno-IncRNA4 homologue
		chr7:164873802-164874792	29	SNORD114, SNORD114	N/A	N/A	N/A
		chr10:15174906-15176653	13	SNORD12B, SNORD12C	ZFAS1	N/A	N/A
		chr19:55523975-55524331	12	SNORD33, SNORD34	RPL13A	N/A	RPL13A region sno-IncRNA homologue
Mouse	ESC (R1)	chr7:52381972-52382315	146	SNORD33, SNORD34	RPL13A	Yes	RPL13A region sno-IncRNA

19 *sno-lncRNAs* identified in this study were listed with their species source, cell line source, genomic locations, expression level (RPKM), snoRNA ends, parent genes, validation and other notes. It includes eight *sno-lncRNAs* in human ESCs, three in ENCODE human cell lines, seven in rhesus ESCs and one in mouse ESCs. Expression levels of *sno-lncRNAs* were evaluated using RPKM in relevant cell lines (the ENCODE GM12878 cell line was used). Due to the poor annotation, host genes and snoRNA ends in rhesus were predicted from human and/or mouse homologous sequences. *one *sno-lncRNA* that contains a box C/D snoRNA at one end and a box H/ACA snoRNA at the other end was highlighted with asterisk.

To further explore *sno-lncRNA* candidates, we developed a custom computational pipeline to predict *sno-lncRNAs* by integrating snoRNA annotations with poly(A)-/ribo- RNA-seq datasets from human [14], rhesus and mouse (GEO:GSE53942) (Figure 1B, Methods). By applying this pipeline to multiple poly(A)-/ribo- RNA-seq datasets, 19 *sno-lncRNAs* were identified from different species and/or different cell lines (Table 1). Two additional *sno-lncRNAs* were predicted in H9 cells and, importantly, both could be validated by Northern blots in human H9, HeLa-J and PA1 cells (Additional file 1). Three more *sno-lncRNAs* were further predicted from ENCODE cell lines (Table 1), suggesting that more *sno-lncRNAs* could be identified when this prediction pipeline is applied to other trancriptomes. Furthermore, it is expected that more *sno-lncRNAs* will be identified after improvements in snoRNA annotation. Interestingly, only one *sno-lncRNA* could be predicted from the entire mouse ESC transcriptomes used in this study (Table 1). This mouse *sno-lncRNA* could be validated by Northern blots from different murine cell lines, as indicated below, but its homolog expression was much lower in rhesus and undetectable in human.

**Figure 2:**
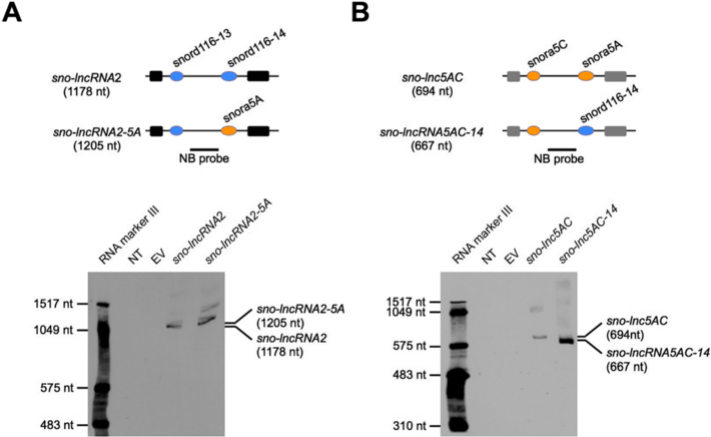
**Either H/ACA or C/D box snoRNP complexes are sufficient for *sno-lncRNA *formation (A) and (B) Northern bolts with denature PAGE gels show that *sno-lncRNAs *can be recapitulated after replacing the snoRNA end from C/D box snoRNA to H/ACA box snoRNA (A) or *vice versa *(B).** Top, a schematic drawing of wild-type *sno-lncRNAs* (*sno-lncRNA2* and *sno-lnc5AC*) or modified *sno-lncRNAs* (*sno-lncRNA2-5A* and *sno-lnc5C-14*) in the expression vector. Black/grey boxes, exons; Black bars, NB probes; Blue circles, C/D snoRNAs; Yellow circles, H/ACA snoRNAs; Bottom, Northern blot validation. NT, no transfection; EV, empty vector. RNA marker III was used to indicate RNA sizes. Total RNAs were separated on 3.5% denature PAGE gels with urea.

Although the majority of predicted *sno-lncRNAs* in human contain either box C/D snoRNAs or box H/ACA snoRNAs on both ends, we found one *sno-lncRNA* from ENCODE datasets that contains a box C/D snoRNA at one end and a box H/ACA snoRNA at the other end (highlighted with asterisk in Table 1). To demonstrate that the hybrid snoRNPs at the ends are also capable of generating *sno-lncRNAs*, we have constructed *sno-lncRNA* expression vectors, which contain a box C/D snoRNA at one end and a box H/ACA snoRNA at the other end to make artificial *sno-lncRNAs* with hybrid snoRNA ends (top panels of Figure 2A and B, Additional file 2A and B, and data not shown). Northern blots clearly demonstrated the successful expression of such *sno-lncRNAs* with hybrid snoRNA ends (bottom panels of Figure 2A and B, Additional file 2A and B, and data not shown). Thus, these data strongly indicate that either box C/D or box H/ACA snoRNP complex at each end is sufficient to protect of internal sequences in *sno-lncRNAs* from nuclease trimming after splicing and that multiple formats of *sno-lncRNAs* may exist in human transcriptomes.

### Low conservation of *sno-lncRNAs* with highly conserved snoRNA ends

**Figure 3:**
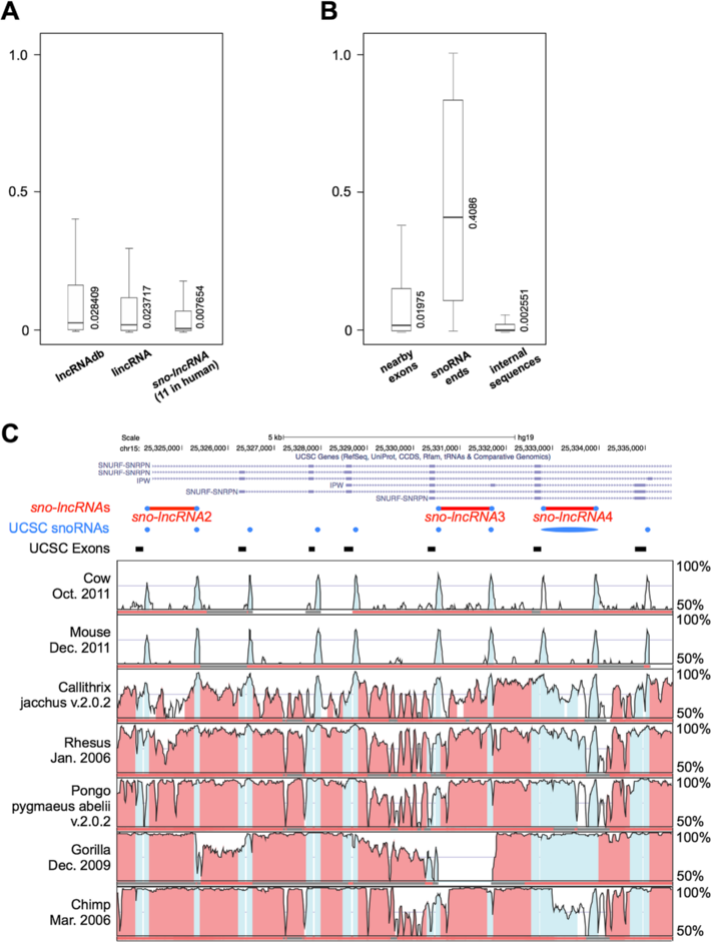
**Highly conserved snoRNA ends from *sno-lncRNAs*. (A)** Conservation analysis of *sno-lncRNAs* and other lncRNAs. *sno-lncRNAs* (11 of human *sno-lncRNAs* summarized in Table 1) are less conserved than other reported lncRNAs [26] or lincRNAs [13]. The median phastCons score was labeled to indicate the average conservation level. **(B)** Conservation analysis of nearby exons, snoRNA ends and internal sequences of *sno-lncRNAs*. snoRNA ends exhibit much higher conservation than nearby exons and internal sequences. The median phastCons score was labeled to indicate the average conservation level. **(C)** Sequence conservation analysis of PWS region in different species with VISTA browser. PWS region snoRNAs (SNORD116 cluster, light blue) exhibit a remarkably higher conservation across species than *SNURF-SNRPN* exons and introns. Y-axis, species selected for comparing (left panel) and conservation levels (right panel); Red bars, human PWS region *sno-lncRNAs*; Blue circles, human PWS region snoRNAs (SNORD116 cluster); Black bars, exons of human PWS region *sno-lncRNA* host gene (*SNURF-SNRPN*). Colors of conserved regions were labeled by VISTA according to UCSC annotations (exons in blue and introns in red). Note that *sno-lncRNA*4 was previously identified as PWCR1 mRNA or small nucleolar RNA (snord116-20) according to UCSC annotation (blue). An analysis of all five PWS region *sno-lncRNAs* and their flanking sequences was highlighted in Additional file 3.

**Figure 4:**
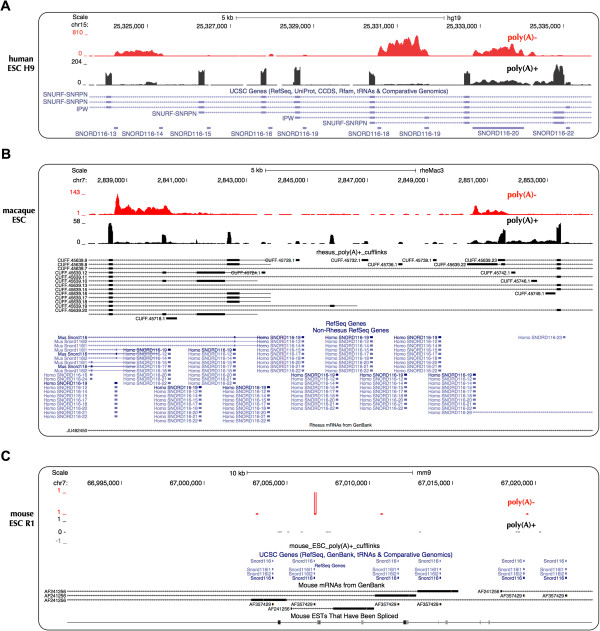
**Unique expression of PWS region *sno-lncRNAs *across species.** Normalized read densities of poly(A)-/ribo- RNA-seq (red) and poly(A)+ RNA-seq (black) from human **(A)**, rhesus **(B)** and mouse **(C)** were shown from UCSC genome browser with customer bigwig inputs. Confident *sno-lncRNA* signals were detected from human and rhesus ESCs, but not from mouse ESCs. Note there is no rhesus Refseq annotation in this regions, transcripts from *de novo* assembly (black lines) and transposed annotations from other species (blue lines) are shown.

Compared to coding genes, lncRNAs are generally expressed at low levels and are not well conserved, which have impeded their discovery and functional analyses (for review see [25]). We examined the sequence conservation of *sno-lncRNAs* by calculating PhastCons scores from multiple alignments of primate genomes. Such analysis revealed that the conservation of *sno-lncRNAs* is the lowest among other well-characterized lncRNAs [26] and predicted lincRNAs [13] (Figure 3A). However, the conservation of snoRNAs themselves is much higher than internal sequences of *sno-lncRNAs* or nearby exons (Figure 3B). For example, PWS region snoRNAs (SNORD116 cluster in human) exhibit a remarkably higher conservation across species than either the SNURF-SNRPN exons or introns (Figure 3C and Additional file 3). However, the high conservation of genomic sequences does not necessarily imply the expression of homologous RNAs at the transcriptome level, as indicated below (Figure 4 and Additional file 4 and Additional file 5). The expression of *sno-lncRNAs* is highly restricted to specific species and no homology could be detected from all three species among all predicted *sno-lncRNAs* (Table 1).

### PWS region *sno-lncRNAs* are highly expressed in human, but undetectable in mouse

The genomic context of the PWS region is complex and the characterization of this region across species is still lacking comprehensive analysis. We first examined the genomic context of PWS region *sno-lncRNAs* by comparing their genomic sequences from different species. Given that *sno-lncRNA* formation depends on snoRNA sequences at both ends within a single intron, the highly conserved PWS region SNORD116 snoRNAs suggested the likelihood of the formation of PWS region *sno-lncRNAs* in other species. Compared to five in human (Figure 4A and Additional file 3), interrogation of poly(A)-/ribo- RNA-seq datasets revealed only two PWS region *sno-lncRNAs* from alternative spliced introns in rhesus ESC cells (Figure 4B and Additional file 4), but none in mouse cells (Figure 4C and Additional file 5). In addition, no clear evidence for PWS region *sno-lncRNAs* could be found in mouse brain/hippocampus in which the *SNURF-SNRPN* transcript and its downstream noncoding region are highly transcribed (Additional file 5), further indicating the absence of PWS region *sno-lncRNAs* in mouse.

SNORD116 snoRNAs are highly conserved from human and rhesus to mouse (Figure 3C and Additional file 3); however, obvious differences in their genomic locations were observed in the PWS region. In human/rhesus genomes, SNORD116s are located in introns of the parent *SNURF-SNRPN* transcript and some of them form snoRNA pairs in one alternative spliced intron, which results in the formation of *sno-lncRNAs* (Figures 4A and B). In the mouse genome, SNORD116s are located in introns of a series of spliced ESTs, which are located at least 50 kb away from the *SNURF-SNRPN* locus (Figure 4C and Additional file 5). Although there are expressed signals of spliced ESTs in the mouse hippocampus transcriptome (Additional file 5), no SNORD116 snoRNA pairs were found between these spliced ESTs, thus no *sno-lncRNAs* could be generated from this region in mouse. Taken together, although snoRNA ends are essential for the formation of *sno-lncRNAs*, the existence of highly-conserved snoRNAs alone is not sufficient for their formation.

The genomic region encoding 15q11-13 *sno-lncRNAs* is specifically deleted in human PWS. PWS is a multiple system disorder with a minimal paternal deletion in chr15 [27]. The deficiency of SNORD116 snoRNAs within the minimal deletion has been thought to play an important role in the pathogenesis of PWS [20-22]. However, mouse models with SNORD116 deletions can only partially mimic PWS phenotypes, including metabolism and growth deficiency, but not obesity [28,29]. Although the mechanism of PWS pathogenesis still remains mysterious, the recent finding of *sno-lncRNAs* in the PWS region in human and their regulatory function in splicing has offered an additional functional layer of gene regulation underlying PWS pathogenesis [15]. The finding of no expression of PWS region *sno-lncRNAs* in mouse indicates a possible limitation of the use of mouse models to study human PWS.

### Characterization of PWS region *sno-lncRNAs* in rhesus revealed that they sequester Fox proteins like human *sno-lncRNAs*

**Figure 5:**
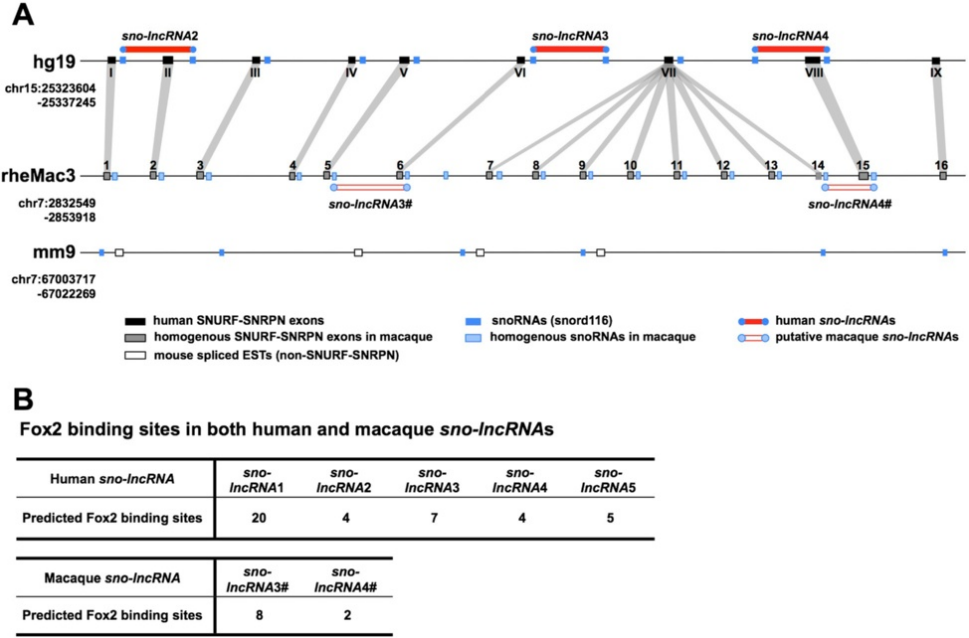
**Distinct landscapes of PWS regions across species. (A)** Comparison of PWS region *sno-lncRNAs* and its parent *SNURF-SNRPN* exons in different species. Conserved *SNURF-SNRPN* exons were manually linked between human (black boxes of top panel) and rhesus (grey boxes of middle panel), and SNORD116 cluster snoRNAs (dark and light blues bars for human and rhesus, respectively) were located in introns. Note that several non-*SNURF-SNRPN* exons were annotated in mouse (empty boxes). The homogenous *SNURF-SNRPN* exons in rhesus are based on sequence homology and/or expression signals (Additional file 6). **(B)** Enrichment of Fox binding sites in PWS region *sno-lncRNAs*. Potential Fox protein binding sites were predicted in five human and two rhesus *sno-lncRNAs*.

We next inspected PWS region *sno-lncRNAs* in rhesus in greater detail. Since the genomic context of the PWS region is complex and the transcriptome annotation in rhesus is limited, we used *SNURF-SNRPN* exons to locate SNORD116 snoRNAs and PWS region *sno-lncRNAs* in rhesus. The rhesus *SNURF-SNRPN* exons were determined by transposing the human/mouse homologous exons (Figure 5A), and putative *SNURF-SNRPN* transcripts could be identified in rhesus by *de novo* assembly from a poly(A)+ RNA-seq dataset (GSE53942). Pair-wise sequence alignments between human (black bars in Figure 5A) and rhesus (grey bars in Figure 5A) suggested that *SNURF-SNRPN* exons exhibit high sequence similarity (Additional file 6). Interestingly, one human *SNURF-SNRPN* exon (the seventh) is repeated at least eight times in the rhesus PWS region; however it is unclear whether this repetitive region occurs only in rhesus or has been lost in humans during evolution.

Sequence alignment of SNORD116 snoRNAs and their parent *SNURF-SNRPN* exons revealed that two PWS region *sno-lncRNAs* in rhesus are similar to human PWS region *sno-lncRNA*3 and *sno-lncRNA*4, respectively. Although predicted rhesus SNORD116 snoRNAs are scattered among individual introns (Figure 5A), *de novo* assembly with rhesus poly(A)+ RNA-seq revealed a variety of alternatively spliced *SNURF-SNRPN* transcripts in rhesus, thus leading to the formation of *sno-lncRNAs* in rhesus *SNURF-SNRPN* region (Figure 4B, transcripts from *de novo* assembly shown in thick black lines).

Human PWS region *sno-lncRNAs* could function as molecular sponges by associating with Fox family splicing regulators and altering patterns of splicing [15]. Due to the high similarity of rhesus PWS region *sno-lncRNAs* with human in the genomic context, we reasoned that they might function similarly as well. We thus scanned the rhesus *sno-lncRNA* sequence for Fox binding motifs, and identified an enrichment of Fox binding sites (Figure 5B), further indicating that rhesus PWS region *sno-lncRNAs* might also interact with Fox family splicing regulators and play a similar role in splicing regulation. On the other hand, the absence of PWS region *sno-lncRNAs* in mouse indicated that a similar regulation mechanism is absent in mouse.

In sum, PWS region *sno-lncRNAs* are highly expressed in human and rhesus, but are absent in mouse. The absence of PWS region *sno-lncRNAs* in mouse also suggests one possible reason to explain the failure of current mouse deletion models to fully recapitulate pathological features of human PWS [27-29]. However, we cannot exclude other regulatory pathways or mechanisms during PWS pathogenesis.

### A non-human *sno-lncRNA* and its possible association with the regulation of lipid toxicity

**Figure 6:**
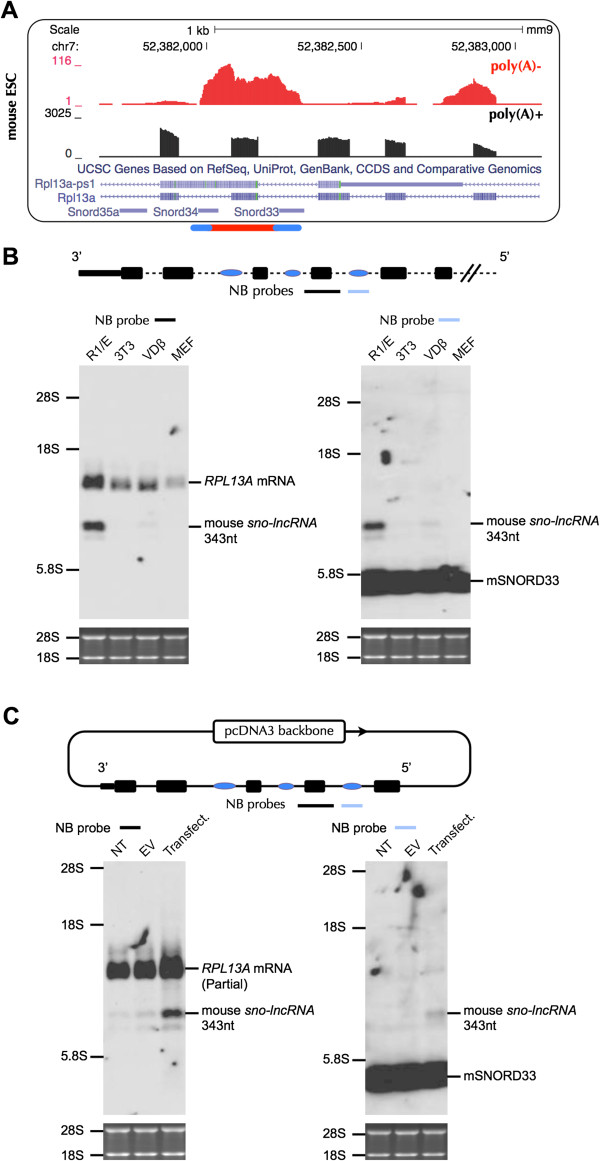
**Expression of a non-human *sno-lncRNA *in *RPL13A *region in mouse. (A)** A highly expressed *sno-lncRNA* was indicated in mouse ESC transcriptomes. Top, normalized read densities of poly(A)-/ribo- (red) and poly(A)+ RNA-seq (black) in mouse ESCs. Note two ends of this *sno-lncRNA* (indicated below) map precisely to the intron-imbedded SNORD33 and SNORD34*.***(B)** Northern blot validation of this *sno-lncRNA* in mouse cell lines. This *sno-lncRNA* was highly expressed in mouse R1 cells, but less expressed in other mouse cell lines (NIH 3 T3, VDβ and MEF) with probes for parent *RPL13A* exon (black bar, left panel) or SNORD33 (blue bar, right panel) by Northern blots. Top, a schematic drawing of *RPL13A* gene (black bars) and intron-embedded snoRNAs (SNORD35, SNORD34 and SNORD33, from left to right, blue bars). Note that the NB probe recognizing SNORD33 can also visualize other mouse snoRNAs due to the sequence similarity of snoRNAs. **(C)** Recapitulation of *RPL13A* region *sno-lncRNA* in NIH 3 T3 cells. Top, a schematic drawing of mouse *sno-lncRNA* flanked by its full length intron and exons in expression vector. Bottom, Northern blots of recapitulated mouse *sno-lncRNA* with probes recognizing different regions. NT, no transfection; EV, empty vector. Note that endogenous *RPL13A* region *sno-lncRNA* is lowly expressed in NIH 3T3 cells.

Although none homologue of human *sno-lncRNAs* identified in this study could be detected in mouse transcriptomes, there is one highly-expressed *sno-lncRNA* predicted in mouse ESCs. This *sno-lncRNA* is flanked by SNORD33 and SNORD34 at its ends and is located in *ribosomal protein L13a* (*RPL13A*) gene (Figure 6A). Northern blots with different probes demonstrated the existence of this mouse *sno-lncRNA* of the expected size in several mouse cell lines with both native agarose gels (Figure 6B) and denatured PAGE gels (Additional file 7). Furthermore, this *RPL13A* region *sno-lncRNA* is highly expressed in mouse ESC cells, but less in other mouse lines (Figure 6B). Moreover, it can be recapitulated in expression vectors, as indicated in Figure 6C. Strikingly, even though both the sequences and structures of snoRNA ends and *RPL13A* exons are highly conserved from human to mouse (Additional file 8), the *sno-lncRNA* was not expressed in examined human cell lines, and expressed at very low levels in rhesus ESCs (Table 1).

SnoRNAs within *RPL13A* introns are critical mediators of lipotoxic cell death in both hamster and mouse [30]. Lipotoxic stress strongly induces expression of these snoRNAs, but has no effect on the steady state levels of the parent *RPL13A* gene [30]. While it is unclear whether *RPL13A* region *sno-lncRNA* is also involved in lipotoxicity like its snoRNA ends, our finding offers another possible regulation for gene expression in this region and it will be of interest to study the function of this *RPL13A* region *sno-lncRNA*.

### Species-specific alternative splicing leads to the formation of the *RPL13A* region *sno-lncRNA* in the mouse

The analyses of PWS region *sno-lncRNAs* and the mouse *RPL13A* region *sno-lncRNA* strongly indicated that *sno-lncRNAs* are expressed in a species-specific manner. To determine whether there are differences in the biogenesis process of *sno-lncRNAs* in different species, we individually transfected expression vectors for mouse *sno-lncRNA* into human cells or expression vectors for human *sno-lncRNA* into mouse cells. Interestingly, both species-specific *sno-lncRNAs* could be recapitulated in cultured cells from other species (Additional file 9), suggesting that species-specificity of *sno-lncRNAs* is mainly derived from their genomic context instead of from the underlying biogenesis machinery.

**Figure 7:**
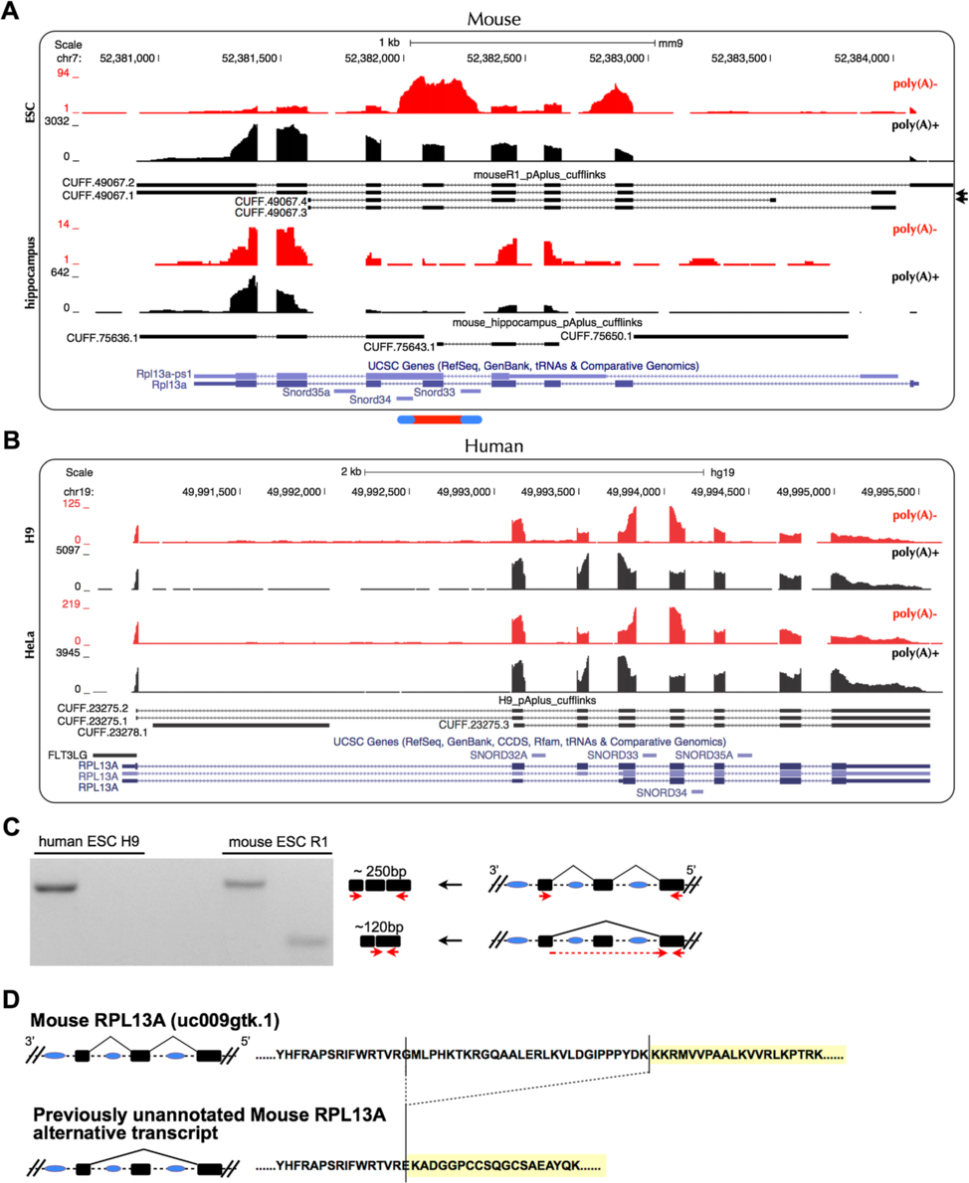
**Species-specific *RPL13A *region *sno-lncRNA *is derived from species-specific alternatively spliced *rpl13a *transcripts. (A)***De novo* transcript assembly revealed previously uncharacterized alternative spliced *rpl13a* transcripts (mouseR1_pAplus_cufflinks). At least two new *rpl13a* isoforms (back arrows on right) were identified to splice out an intron containing SNORD33 and SNORD34. Y-axis, normalized read densities of poly(A)-/ribo- RNA-seq (red) and poly(A)+ RNA-seq (black) of mouse ESC and hippocampus transcriptomes. **(B)** No such alternative spliced *rpl13a* transcripts in human ESC H9 and HeLa cells from both *de novo* assembly (h9_pAplus_cufflinks) and known annotation (blue lines). Y-axis, normalized read densities of poly(A)-/ribo- RNA-seq (red) and poly(A)+ RNA-seq (black) of human H9 and HeLa transcriptomes. **(C)** Validation of alternative spliced *rpl13a* transcripts by RT-PCR. Left, the previously uncharacterized alternative splicing event of *RPL13A* gene could only be detected in mouse, but not in human, with RT-PCR. Right, a schematic drawing of different splicing events and their validation primers in *RPL13A*. Black bars, *RPL13A* exons; Blue circles, snoRNAs; Red arrows, PCR primer sets. **(D)** The alternative splicing of *RPL13A* causes amino acid changes at the C-terminal of RPL13A protein. Top, schematic diagram of canonical splicing of *RPL13A* and its amino acid sequence; Bottom, schematic diagram of uncharacterized splicing of *RPL13A* and its amino acid sequence.

As mentioned above, snoRNAs generally are located within introns, but most of them do not exist as pairs in single introns (Figure 1A). With known intron annotations, only a few *sno-lncRNAs* could be identified (Figure 1A). However, using an intron-annotation-independent *sno-lncRNA* prediction strategy (Figure 1B), several additional novel *sno-lncRNAs* were identified in examined transcriptomes (Table 1), suggesting that previously uncharacterized alternative splicing events can generate snoRNAs pairs within one intron. For example, the PWS region *sno-lncRNAs* in rhesus could be generated from alternative spliced *SNURF-SNRPN* (Figure 4B). Taking the *RPL13A* region *sno-lncRNA* into consideration, the gene organization of *RPL13A* is highly conserved across species and SNORD33 and SNORD34 are usually confined to distinct single introns. Given the fact that only two snoRNA genes located within one intron can lead to the formation of a *sno-lncRNA*, this suggests that the existence of a previously uncharacterized alternative splicing event in the adjacent introns of SNORD33 and SNORD34. To test this possibility, we did *de novo* transcript assembly with a poly(A)+ RNA-seq dataset, and successfully identified the alternative splicing event that results in the location of SNORD33 and SNORD34 into one intron (Figure 7A). Interestingly, a similar alternative splicing event could be assembled in rhesus (Additional file 10), but not in human (Figure 7B), which is consistent with the lack of an expression signal for a *RPL13A* region *sno-lncRNA* in human. In addition, RT-PCR results further confirmed the alternative splicing in mouse, but none in human (Figure 7C). Thus, the alternative splicing of *RPL13A* gene in mouse leads to the formation of a species-specific *sno-lncRNA*. On the other hand, the lack of *RPL13A* alternative splicing in human likely prevents the expression of this *sno-lncRNA*. It should be noted that there is low expression of *RPL13A* region *sno-lncRNA* in rhesus, together with a low level of the necessary alternative splicing (Additional file 10).

Very interestingly, further analyses revealed that the specific *RPL13A* alternative splicing event that leads to the production of *sno-lncRNA* could also generate a protein of altered amino acid sequence in both mouse (Figure 7D) and rhesus (data not shown), although the change of protein sequence and its consequence need to be further experimentally confirmed. As a 60S ribosomal subunit protein, RPL13A is highly conserved across species and plays an essential role in protein synthesis. Therefore, the finding of alternative splicing of *RPL13A* has implicated a possible role in the regulation of 60S ribosome assembly or function in a species-specific manner. Since a large diversification of splicing exists between tissues and species [31,32] and *sno-lncRNAs* are expressed with tissue- and species-specific patterns, it quite likely that more such RNAs will be uncovered when additional tissues and species samples are examined. Taken together, alternative splicing not only increases the diversity of coding mRNAs/proteins, but also expands transcriptome complexity by promoting the formation of noncoding RNAs from untranslated intron sequences.

## Conclusion

We explored non-polyadenylated transcriptomes (poly(A)-/ribo-) from human, rhesus and mouse, and systematically annotated *sno-lncRNAs* across species. Although primary sequence analysis revealed that snoRNA ends of such molecules are highly conserved, PWS region *sno-lncRNAs* are highly expressed in human and rhesus, but absent in mouse. The absence of PWS region *sno-lncRNAs* in mouse suggested a possible reason for the failure of the current mouse model to fully recapitulate pathological features of human PWS. Only one mouse *sno-lncRNA* was identified from the limited available mouse datasets in *RPL13A* region, and snoRNAs themselves in this region have been suggested to be involved in lipotoxicity in mouse. Our results also demonstrated that the formation of *sno-lncRNAs* often requires alternative splicing within their parent genes, indicating a complex regulatory network of coding and noncoding parts of the genome.

## Methods

### Annotation of snoRNAs across species

Annotated human snoRNAs derived from snoRNABase [24] (https://www-snorna.biotoul.fr/) were downloaded from UCSC Genome Bioinformatics database (http://hgdownload.soe.ucsc.edu/goldenPath/hg19/database/wgRna.txt.gz, updated on 2010/10/3). 132 mouse snoRNA annotations were downloaded from RefSeq database (http://www.ncbi.nlm.nih.gov/refseq/, downloaded on 2013/3/4). 375 putative rhesus snoRNAs were transposed from human and mouse snoRNA annotations using liftOver (http://genome.ucsc.edu/cgi-bin/hgLiftOver) with minMatch = 0.95 and combined together to be used as rhesus snoRNA annotations. All these snoRNA annotations were overlapped respectively with relevant gene annotations according to their species-derivation (Human: UCSC Genes, updated on 2012/2/5; Rhesus: RefSeq Genes, updated on 2013/3/24; Mouse: UCSC Genes, updated on 2011/5/30) to find snoRNA pairs (at least two snoRNAs in one intron) in introns, as indicated in Figure 1A. SnoRNA pairs in the same introns were further examined from poly(A)-/ribo- RNA-seq datasets to identify putative *sno-lncRNAs*.

### Sequencing read alignment and transcript *de novo* assembly

The poly(A)+ or poly(A)-/ribo- RNA-seq reads were uniquely aligned to relevant genomes (Human: hg19, GRCh37; Rhesus: rheMac3, BGI CR_1.0; Mouse: mm9, NCBI37) using TopHat 2.0.8 [33] (parameters: -g 1 -a 6 -i 50 --microexon-search --coverage-search -m 2) with existing annotations (Human: UCSC Genes, updated on 2012/2/5; Rhesus: RefSeq Genes, updated on 2013/3/24; Mouse: UCSC Genes, updated on 2011/5/30), respectively. To facilitate the identification of potential *sno-lncRNAs*, Bowtie 0.12.9 [34] (parameters: -v 3 -k 1 -m 1) was also employed to map poly(A)-/ribo- RNA-seq reads to annotated genome references. Expression level (RPKM) of annotated genes (including snoRNAs) was obtained with customized pipeline (Zhu *et al.*, in preparation). Cufflinks v2.0.2 [35] (parameters: -F 0) was employed to assemble poly(A)+ RNA-seq mapping results to obtain *de novo* RNA transcripts. All mapping results were normalized and uploaded to the UCSC Genome Browser (http://genome.ucsc.edu/) for visualization.

### Computational pipeline to identify *sno-lncRNAs* from poly(A)-/ribo- RNA-seq datasets

To systematically identify *sno-lncRNAs* independently from known gene annotation, we developed a custom *sno-lncRNA* identification pipeline (termed as SNOLNCfinder), as indicated in Figure 1B. Briefly, for each two adjacent snoRNAs (distance < 10 kb), RPKMs of snoRNA regions and their internal regions were calculated with a customized pipeline (Zhu *et al.*, in preparation) from Bowtie mapped poly(A)-/ribo- RNA-seq reads. A putative *sno-lncRNA* was selected with 1) both snoRNA pairs are expressed with RPKM ≥ 1; 2) at least 80% of the internal region between snoRNA pairs have poly(A)-/ribo- RNA-seq signals by sliding window examination (Figure 1B); and 3) relatively high expression of the internal region (at least 40% of expression of snoRNA pairs). All candidates were manually inspected by comparing the poly(A)+ and poly(A)-/ribo- RNA-seq datasets. This pipeline is independent on known gene annotation and can be successfully employed in human, rhesus and mouse datasets to identify new *sno-lncRNAs*. RNA-seq datasets used here were from human ESC H9 cells and HeLa cells(GEO:GSE24399), ENCODE cell lines (GEO: GSE26284). RNA-seq files for rhesus ESCs, mouse ESCs and mouse hippocampus can be accessed from the NCBI Sequence Read Archive by Gene Expression Ominbus accession number (GEO:GSE53942).

### Genomic sequence comparison with VISTA

VISTA Browser [36] (http://genome.lbl.gov/vista/) was employed to inspect the conservation landscape for a given region from different genomes, including human (Feb. 2009), Cow (Oct. 2011), Mouse (Dec. 2011 or Jul. 2007), Callithrix jacchus v.2.0.2 (Jun. 2007), Rhesus (Jan. 2006), Pongo pygmaeus abelii v.2.0.2 (Jul. 2007), Gorilla (Dec. 2009), Chimp (Mar. 2006), Rat (Nov. 2004), Dog (May 2005) and Horse (Jan. 2007).

### *SNURF-SNRPN* sequence comparative analysis

Putative rhesus snoRNA116s (Figure 4B) were marked with human and mouse annotations (Figures 4A and C) in UCSC. Locations of putative rhesus *SNURF-SNRPN* exons were defined according to rhesus poly(A)+ RNA-seq mapping signals. Sequences of human (Figure 4A) and rhesus (Figure 4B) *SNURF-SNRPN* exons were extracted from UCSC Genome Bioinformatics database (http://genome.ucsc.edu/). Pair-wise sequence alignments were carried out using T-Coffee [37].

### Fox protein binding site prediction on PWS *sno-lncRNAs*

Sequences of human PWS *sno-lncRNAs* and rhesus putative PWS *sno-lncRNAs* were extracted from UCSC Genome Bioinformatics database (http://genome.ucsc.edu/). All these sequences were scanned for Fox hexanucleotide motifs including UGCAUG, GCAUGU, GUGAUG, UGGUGA and GGUGGU [38].

### Conservation analysis with PhastCons

PhastCons scores for multiple alignments of primate genomes (http://hgdownload.cse.ucsc.edu/goldenpath/hg19/database/phastCons46wayPrimates.txt.gz, updated on 2009/12/6) were downloaded from UCSC and corresponding PhastCons scores for lncRNAs [26], lincRNAs [13] and *sno-lncRNAs* (11 predicted in human, Table 1) were counted separately to inspect the conservation difference of these three datasets. PhastCons scores for nearby exons, snoRNAs at both ends of *sno-lncRNAs* and internal regions of *sno-lncRNAs* were also calculated separately to investigate the region-specific conservation difference of *sno-lncRNAs*.

### Cell culture, cell transfection and antisense oligonucleotide treatment

All cell lines were cultured using standard protocols. Plasmid transfection was carried out with X-tremeGENE 9 (Roche) or with nucleofection (Lonza) according to the manufacturer’s instructions. Rhesus rhesus RNAs were extracted from ESC line IVF3.2 [39]. Mouse RNAs were extracted from ESC R1 line or sacrificed mouse hippocampus, respectively. Mice were maintained and used in accordance with the guidelines of the Institutional Animal Care and Use Committee of Shanghai Institutes for Biological Sciences.

### Plasmids construction

SNORD116-14 in pcDNA3-*sno-lncRNA*2 [15] was replaced with SNORA5A to generate construct pcDNA3-*sno-lncRNA2-5A* (Figure 2A), and SNORA5A in pcDNA3-*sno-lnc5AC* was substituted with SNORD116-14 to generate constructs pcDNA3-*sno-lnc5C-14* (Figure 2B) with primers listed in Table S1. Mouse *sno-lnc33/34* and human *sno-lnc5AC* flanked by its full length intron, splice sites and exons were cloned into pcDNA3 (Figure 6C).

### RNA Isolation, poly(A)-/ribo- fractionation, RNA-seq and Northern Blot

Cultured cell lines or cells with different treatments were harvested in Trizol (Invitrogen) and RNAs were extracted according to the manufacturer’s instruction, followed by DNase I treatment at 37°C for 30 mins (Ambion, DNA-free™ Kit). Poly(A)+ and poly(A)-/ribo- RNA transcripts were fractionated and sequenced as previously described [15]. Raw sequencing dataset and bigWig track file of rhesus and mouse poly(A)-/ribo- RNAs are available for download from NCBI Gene Expression Omnibus under accession number GSE53942 for mouse ESCs, mouse hippocampus and rhesus ESCs. Northern Blot was carried out according to the manufacturer’s protocol (DIG Northern Starter Kit, Roche). Denatured RNAs were loaded on either native agarose gel or denatured PAGE gel with urea for Northern Blots as previous studies [15,16]. Digoxigenin (Dig) labeled antisense and sense probes were made using either SP6 or T7 RNA polymerase by *in vitro* transcription with the AmpliScribe™ SP6 and T7 High Yield Transcription Kits (Epicentre). DIG-labeledRNA Molecular Wight Marker III is from Roche.

## Competing interests

The authors declare that they have no competing interests.

## Authors’ contributions

LY and LLC designed the project. XOZ and LY performed bioinformatics analyses. QFY, HBW, YZ, TC, PZ and XL performed experiments. LY, LLC and XOZ analyzed the data. LY and LLC wrote the paper with inputs from other authors. All authors read and approved the final manuscript.

## Supplementary Material

Additional file 1 **Identification and validation of two novel human *sno-lncRNAs.*** (A) Expression patterns of predicted *sno-lncRNA* in human cell lines. Normalized read densities of poly(A)-/ribo- RNA-seq (red) and poly(A)+ RNA-seq (black) were indicated in H9 and HeLa, respectively. Red bar, NB probe for (B). (B) Northern blot validation of this novel *sno-lncRNA* (~598 nt) in H9, PA-1 and HeLa-J cell lines. (C) Expression patterns of predicted *sno-lncRNA* in human cell lines. Left, normalized read densities of poly(A)-/ribo- RNA-seq (red) and poly(A)+ RNA-seq (black) were indicated in H9 and HeLa, respectively. Red bar, NB probe for (B). (D) NB validation of this novel *sno-lncRNA* (~585 nt) in H9, PA-1 and HeLa-J cell lines. Click here for file

Additional file 2 **Northern blots of *sno-lncRNAs *with native agarose gel.** (A) and (B) Northern bolts show that *sno-lncRNAs* can be recapitulated after replacing the snoRNA end from C/D box snoRNA to H/ACA box snoRNA (A) or *vice versa* (B). Top, a schematic drawing of wild-type *sno-lncRNAs* (*sno-lncRNA2* and *sno-lnc5AC*) or modified *sno-lncRNAs* (*sno-lncRNA2-5A* and *sno-lnc5C-14*) in the expression vector. Black/grey boxes, exons; Black bars, NB probes; Blue circles, C/D snoRNAs; Yellow circles, H/ACA snoRNAs; Bottom, Northern blot validation. NT, no transfection; EV, empty vector. RNA marker III was used to indicate RNA sizes. Denatured RNAs were separated on 1% agarose gel. Note that similar RNA separations were obtained by both denatured PAGE gels (Figure 2) and native (shown here) agarose gels. Click here for file

Additional file 3 **Sequence conservation analysis of PWS region across species.** PWS region snoRNAs (SNORD116 cluster snoRNAs, light blue) exhibit a remarkably higher conservation across species than *SNURF-SNRPN* exons and introns. Y-axis, species selected for comparing (left panel) and conservation levels (right panel); Red bars, human PWS region *sno-lncRNAs*; Blue circles, human PWS region snoRNAs (SNORD116 cluster); Black bars, exons of human PWS region *sno-lncRNA* host gene (*SNURF-SNRPN*). Click here for file

Additional file 4 **Expression of PWS region in rhesus.** Normalized read densities of poly(A)-/ribo- RNA-seq (red) and poly(A)+ RNA-seq (black) in rhesus ESCs showed two highly expressed *sno-lncRNAs* in PWS region. Note that there is no RefGene annotation in rhesus, instead, homologues genes from other species are shown. Click here for file

Additional file 5 **Expression of PWS region in mouse.** Normalized read densities of poly(A)-/ribo- RNA-seq (red) and poly(A)+ RNA-seq (black) of PWS region in mouse ESC R1 and mouse hippocampus showed undetected expression of PWS region *sno-lncRNAs*. Note that mouse SNORD116 snoRNAs are over 50 kb away from mouse *SNURF-SNRPN*. These SNORD116 snoRNAs and their adjacent spliced ESTs are not expressed in mESCs, but are expressed in mouse hippocampus. Click here for file

Additional file 6 **Pair-wise sequence alignments of *SNURF-SNRPN *exons between human (black bars of Figure** 5**A) and rhesus (grey bars of Figure** 5**A).** Click here for file

Additional file 7 **Northern blot of mouse-specific *sno-lncRNA *in *RPL13A *region.** Northern blot validation of mouse specific *sno-lncRNA* from multiple mouse cell lines. Total RNAs from ESC R1, NIH 3T3, VDβ and MEF were denatured and separated on 8% denatured PAGE gel. After separation, the gel was stained with ethidium bromide for rRNA/tRNA visualization (A), and transferred to membrane for Northern blot with probe for SNORD33 (blue bar of Figure 6B) after destaining (B). Positions for 5.8S, 5S rRNA, and tRNAs were indicated with ethidium bromide staining. Click here for file

Additional file 8 **Sequence conservation analysis of *RPL13A *region *sno-lncRNA.*** Y-axis, species selected for comparing (left panel) and conservation levels (right panel); Red bars, a non-human *sno-lncRNA*; Blue circles, mouse snoRNAs (SNORD35, SNORD34, SNORD33 and SNORD32a, from left to right); Black bars, exons of the non-human *sno-lncRNA* host gene (*RPL13A*). Click here for file

Additional file 9 **Transfection of species-specific *sno-lncRNA *into cell lines derived from different species.** (A) Transfection of human *sno-lncRNA* ito mouse NIH 3T3 cell line generates the human *sno-lncRNA* as revealed by NB. NT, no transfection; EV, empty vector. (B) Transfection of mouse *sno-lncRNA* to human HeLa-J cell line generates the mouse *sno-lncRNA* as revealed by NB. NT, no transfection; EV, empty vector. Click here for file

Additional file 10 **Species-specific *RPL13A *region *sno-lncRNA *is derived from species-specific alternative spliced *rpl13a *transcripts in rhesus.***De novo* transcript assembly revealed previously uncharacterized alternative spliced *rpl13a* transcripts (rhesus_pAplus_cufflinks). One new rhesus *rpl13a* isoform (indicated by arrow) was identified to splice out a large intron containing SNORD33 and SNORD34. Y-axis, normalized read densities of poly(A)-/ribo- RNA-seq (red) and poly(A)+ RNA-seq (black) of rhesus ESC transcriptomes. Click here for file

## References

[B1] International Human Genome Sequencing Consortium Finishing the euchromatic sequence of the human genome Nature 2004 431 931 945 10.1038/nature03001 15496913

[B2] International Human Genome Sequencing Consortium Initial sequencing and analysis of the human genome Nature 2001 409 860 921 10.1038/35057062 11237011

[B3] The ENCODE Project Consortium Identification and analysis of functional elements in 1% of the human genome by the ENCODE pilot project Nature 2007 447 799 816 10.1038/nature05874 17571346 PMC2212820

[B4] Clark MB Amaral PP Schlesinger FJ Dinger ME Taft RJ Rinn JL Ponting CP Stadler PF Morris KV Morillon A Rozowsky JS Gerstein MB Wahlestedt C Hayashizaki Y Carninci P Gingeras TR Mattick JS The reality of pervasive transcription PLoS Biol 2011 9 e1000625 discussion e1001102 10.1371/journal.pbio.1000625 21765801 PMC3134446

[B5] Yamada K Lim J Dale JM Chen H Shinn P Palm CJ Southwick AM Wu HC Kim C Nguyen M Pham P Cheuk S Karlin-Newmann G Liu SX Lam B Sakano H Wu T Yu G Miranda M Quach HL Tripp M Chang CH Lee JM Toriumi M Chan MMH Tang CC Onodera CS Deng JM Akiyama K Ansari Y Empirical analysis of transcriptional activity in the Arabidopsis genome Science 2003 302 842 846 10.1126/science.1088305 14593172

[B6] Pennisi E Shining a light on the genome’s ‘dark matter’ Science 2010 330 1614 10.1126/science.330.6011.1614 21163986

[B7] Landgraf P Rusu M Sheridan R Sewer A Iovino N Aravin A Pfeffer S Rice A Kamphorst AO Landthaler M Lin M Socci ND Hermida L Fulci V Chiaretti S Foa R Schliwka J Fuchs U Novosel A Muller RU Schermer B Bissels U Inman J Phan Q Chien M Weir DB Choksi R Vita GD Frezzetti D Trompeter HI A mammalian microRNA expression atlas based on small RNA library sequencing Cell 2007 129 1401 1414 10.1016/j.cell.2007.04.040 17604727 PMC2681231

[B8] Bartel DP MicroRNAs: target recognition and regulatory functions Cell 2009 136 215 233 10.1016/j.cell.2009.01.002 19167326 PMC3794896

[B9] Djebali S Davis CA Merkel A Dobin A Lassmann T Mortazavi A Tanzer A Lagarde J Lin W Schlesinger F Xue C Marinov GK Khatun J Williams BA Zaleski C Rozowsky J Roder M Kokocinski F Abdelhamid RF Alioto T Antoshechkin I Baer MT Bar NS Batut P Bell K Bell I Chakrabortty S Chen X Chrast J Curado J Landscape of transcription in human cells Nature 2012 489 101 108 10.1038/nature11233 22955620 PMC3684276

[B10] Derrien T Johnson R Bussotti G Tanzer A Djebali S Tilgner H Guernec G Martin D Merkel A Knowles DG Lagarde J Veeravalli L Ruan X Ruan Y Lassmann T Carninci P Brown JB Lipovich L Gonzalez JM Thomas M Davis CA Shiekhattar R Gingeras TR Hubbard TJ Notredame C Harrow J Guigo R The GENCODE v7 catalog of human long noncoding RNAs: analysis of their gene structure, evolution, and expression Genome Res 2012 22 1775 1789 10.1101/gr.132159.111 22955988 PMC3431493

[B11] Guttman M Amit I Garber M French C Lin MF Feldser D Huarte M Zuk O Carey BW Cassady JP Cabili MN Jaenisch R Mikkelsen TS Jacks T Hacohen N Bernstein BE Kellis M Regev A Rinn JL Lander ES Chromatin signature reveals over a thousand highly conserved large non-coding RNAs in mammals Nature 2009 458 223 227 10.1038/nature07672 19182780 PMC2754849

[B12] Ulitsky I Shkumatava A Jan CH Sive H Bartel DP Conserved function of lincRNAs in vertebrate embryonic development despite rapid sequence evolution Cell 2011 147 1537 1550 10.1016/j.cell.2011.11.055 22196729 PMC3376356

[B13] Cabili MN Trapnell C Goff L Koziol M Tazon-Vega B Regev A Rinn JL Integrative annotation of human large intergenic noncoding RNAs reveals global properties and specific subclasses Genes Dev 2011 25 1915 1927 10.1101/gad.17446611 21890647 PMC3185964

[B14] Yang L Duff MO Graveley BR Carmichael GG Chen LL Genomewide characterization of non-polyadenylated RNAs Genome Biol 2011 12 R16 10.1186/gb-2011-12-2-r16 21324177 PMC3188798

[B15] Yin QF Yang L Zhang Y Xiang JF Wu YW Carmichael GG Chen LL Long noncoding RNAs with snoRNA ends Mol Cell 2012 48 219 230 10.1016/j.molcel.2012.07.033 22959273

[B16] Zhang Y Zhang XO Chen T Xiang JF Yin QF Xing YH Zhu S Yang L Chen LL Circular intronic long noncoding RNAs Mol Cell 2013 51 792 806 10.1016/j.molcel.2013.08.017 24035497

[B17] Gardner EJ Nizami ZF Talbot CC Jr Gall JG Stable intronic sequence RNA (sisRNA), a new class of noncoding RNA from the oocyte nucleus of Xenopus tropicalis Genes Dev 2012 26 2550 2559 10.1101/gad.202184.112 23154985 PMC3505824

[B18] Cavaille J Buiting K Kiefmann M Lalande M Brannan CI Horsthemke B Bachellerie JP Brosius J Huttenhofer A Identification of brain-specific and imprinted small nucleolar RNA genes exhibiting an unusual genomic organization Proc Natl Acad Sci U S A 2000 97 14311 14316 10.1073/pnas.250426397 11106375 PMC18915

[B19] Runte MHA Gross S Kiefmann M Horsthemke B Buiting K The IC-SNURF-SNRPN transcript serves as a host for multiple small nucleolar RNA species and as an antisense RNA for UBE3A Hum Mol Genet 2001 10 2687 2700 10.1093/hmg/10.23.2687 11726556

[B20] Sahoo T del Gaudio D German JR Shinawi M Peters SU Person RE Garnica A Cheung SW Beaudet AL Prader-Willi phenotype caused by paternal deficiency for the HBII-85 C/D box small nucleolar RNA cluster Nat Genet 2008 40 719 721 10.1038/ng.158 18500341 PMC2705197

[B21] de Smith AJ Purmann C Walters RG Ellis RJ Holder SE Van Haelst MM Brady AF Fairbrother UL Dattani M Keogh JM Henning E Yeo GSH O’Rahilly S Froguel P Farooqi S Blakemore AIF A deletion of the HBII-85 class of small nucleolar RNAs (snoRNAs) is associated with hyperphagia, obesity and hypogonadism Hum Mol Genet 2009 18 3257 3265 10.1093/hmg/ddp263 19498035 PMC2722987

[B22] Duker AL Ballif BC Bawle EV Person RE Mahadevan S Alliman S Thompson R Traylor R Bejjani BA Shaffer LG Rosenfeld JA Lamb AN Sahoo T Paternally inherited microdeletion at 15q11.2 confirms a significant role for the SNORD116 C/D box snoRNA cluster in Prader-Willi syndrome Eur J Hum Genet 2010 18 1196 1201 10.1038/ejhg.2010.102 20588305 PMC2987474

[B23] Filipowicz W Pogacic V Biogenesis of small nucleolar ribonucleoproteins Curr Opin Cell Biol 2002 14 319 327 10.1016/S0955-0674(02)00334-4 12067654

[B24] Lestrade L Weber MJ snoRNA-LBME-db, a comprehensive database of human H/ACA and C/D box snoRNAs Nucleic Acids Res 2006 34 D158 D162 10.1093/nar/gkj002 16381836 PMC1347365

[B25] Zhu S Zhang X-O Yang L Panning for long noncoding RNAs Biomolecules 2013 2 226 24970166 10.3390/biom3010226 PMC4030883

[B26] Amaral PP Clark MB Gascoigne DK Dinger ME Mattick JS lncRNAdb: a reference database for long noncoding RNAs Nucleic Acids Res 2011 39 D146 D151 10.1093/nar/gkq1138 21112873 PMC3013714

[B27] Cassidy SB Schwartz S Miller JL Driscoll DJ Prader-Willi syndrome Genet Med 2012 14 10 26 10.1038/gim.0b013e31822bead0 22237428

[B28] Skryabin BV Gubar LV Seeger B Pfeiffer J Handel S Robeck T Karpova E Rozhdestvensky TS Brosius J Deletion of the MBII-85 snoRNA gene cluster in mice results in postnatal growth retardation PLoS Genet 2007 3 e235 10.1371/journal.pgen.0030235 18166085 PMC2323313

[B29] Ding F Li HH Zhang S Solomon NM Camper SA Cohen P Francke U SnoRNA Snord116 (Pwcr1/MBII-85) deletion causes growth deficiency and hyperphagia in mice PLoS One 2008 3 e1709 10.1371/journal.pone.0001709 18320030 PMC2248623

[B30] Michel CI Holley CL Scruggs BS Sidhu R Brookheart RT Listenberger LL Behlke MA Ory DS Schaffer JE Small nucleolar RNAs U32a, U33, and U35a are critical mediators of metabolic stress Cell Metab 2011 14 33 44 10.1016/j.cmet.2011.04.009 21723502 PMC3138526

[B31] Barbosa-Morais NL Irimia M Pan Q Xiong HY Gueroussov S Lee LJ Slobodeniuc V Kutter C Watt S Colak R Kim T Misquitta-Ali CM Wilson MD Kim PM Odom DT Frey BJ Blencowe BJ The evolutionary landscape of alternative splicing in vertebrate species Science 2012 338 1587 1593 10.1126/science.1230612 23258890

[B32] Merkin J Russell C Chen P Burge CB Evolutionary dynamics of gene and isoform regulation in Mammalian tissues Science 2012 338 1593 1599 10.1126/science.1228186 23258891 PMC3568499

[B33] Kim D Pertea G Trapnell C Pimentel H Kelley R Salzberg SL TopHat2: accurate alignment of transcriptomes in the presence of insertions, deletions and gene fusions Genome Biol 2013 14 R36 10.1186/gb-2013-14-4-r36 23618408 PMC4053844

[B34] Langmead B Trapnell C Pop M Salzberg SL Ultrafast and memory-efficient alignment of short DNA sequences to the human genome Genome Biol 2009 10 R25 10.1186/gb-2009-10-3-r25 19261174 PMC2690996

[B35] Trapnell C Hendrickson DG Sauvageau M Goff L Rinn JL Pachter L Differential analysis of gene regulation at transcript resolution with RNA-seq Nat Biotech 2013 31 46 53 10.1038/nbt.2450 PMC3869392 23222703

[B36] Frazer KA Pachter L Poliakov A Rubin EM Dubchak I VISTA: computational tools for comparative genomics Nucleic Acids Res 2004 32 W273 W279 10.1093/nar/gkh458 15215394 PMC441596

[B37] Notredame C Higgins DG Heringa J T-Coffee: a novel method for fast and accurate multiple sequence alignment J Mol Biol 2000 302 205 217 10.1006/jmbi.2000.4042 10964570

[B38] Yeo GW Coufal NG Liang TY Peng GE Fu XD Gage FH An RNA code for the FOX2 splicing regulator revealed by mapping RNA-protein interactions in stem cells Nat Struct Mol Biol 2009 16 130 137 10.1038/nsmb.1545 19136955 PMC2735254

[B39] Sun Z Wei Q Zhang Y He X Ji W Su B MicroRNA profiling of rhesus macaque embryonic stem cells BMC Genomics 2011 12 276 10.1186/1471-2164-12-276 21627802 PMC3117859

