# Supplementary figures and images for: Species-specific alternative splicing leads to unique expression of sno-lncRNAs
Source: BMC Genomics. 2014 Apr 16;15:287. doi: 10.1186/1471-2164-15-287 (PMC4234469; doi:10.1186/1471-2164-15-287)

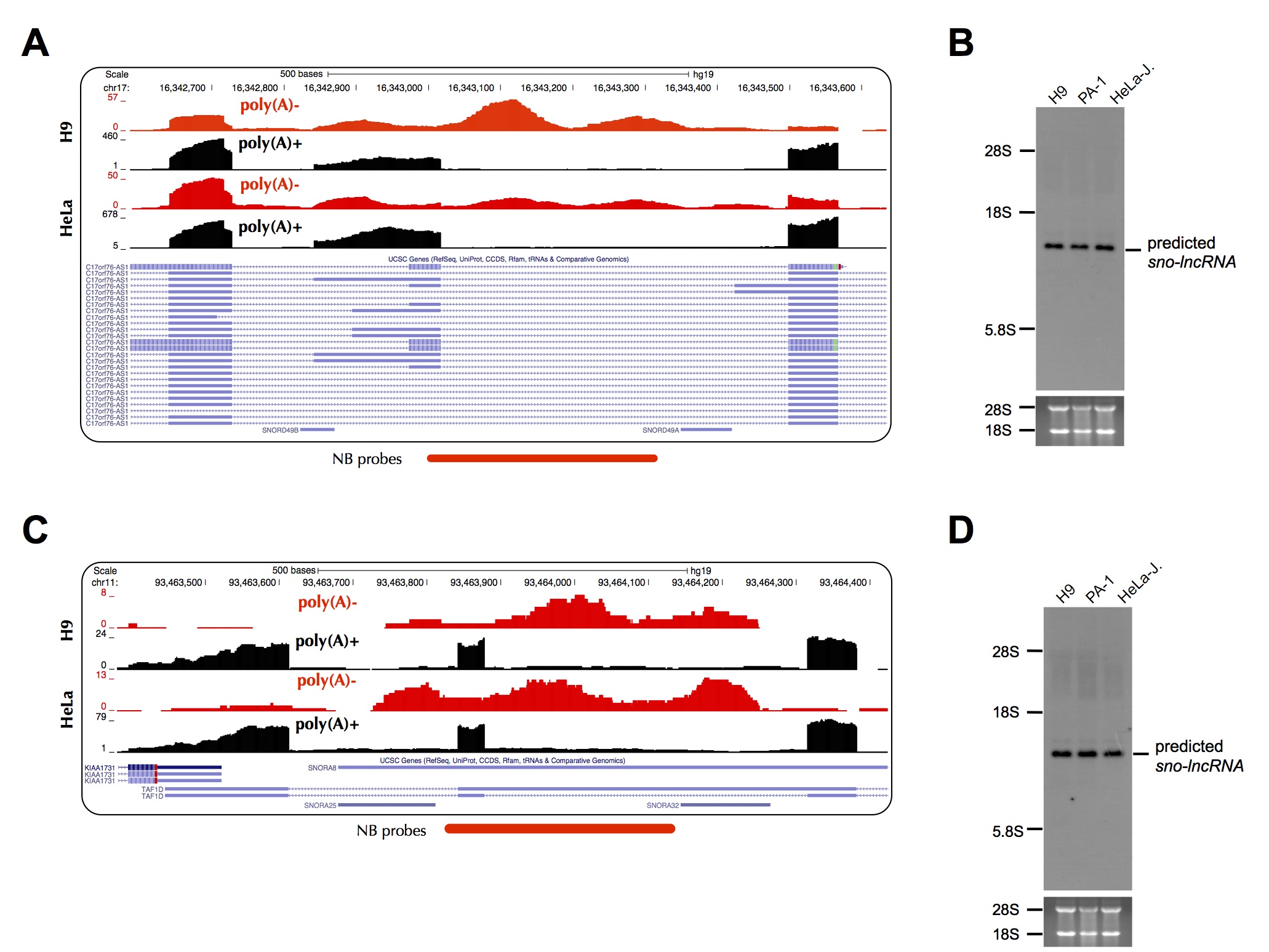

Supplement: Additional file 1 — Identification and validation of two novel human sno-lncRNAs. (A) Expression patterns of predicted sno-lncRNA in human cell lines. Normalized read densities of poly(A)-/ribo- RNA-seq (red) and poly(A)+ RNA-seq (black) were indicated in H9 and HeLa, respectively. Red bar, NB probe for (B). (B) Northern blot validation of this novel sno-lncRNA (~598 nt) in H9, PA-1 and HeLa-J cell lines. (C) Expression patterns of predicted sno-lncRNA in human cell lines. Left, normalized read densities of poly(A)-/ribo- RNA-seq (red) and poly(A)+ RNA-seq (black) were indicated in H9 and HeLa, respectively. Red bar, NB probe for (B). (D) NB validation of this novel sno-lncRNA (~585 nt) in H9, PA-1 and HeLa-J cell lines. [file 1471-2164-15-287-S1.jpeg]

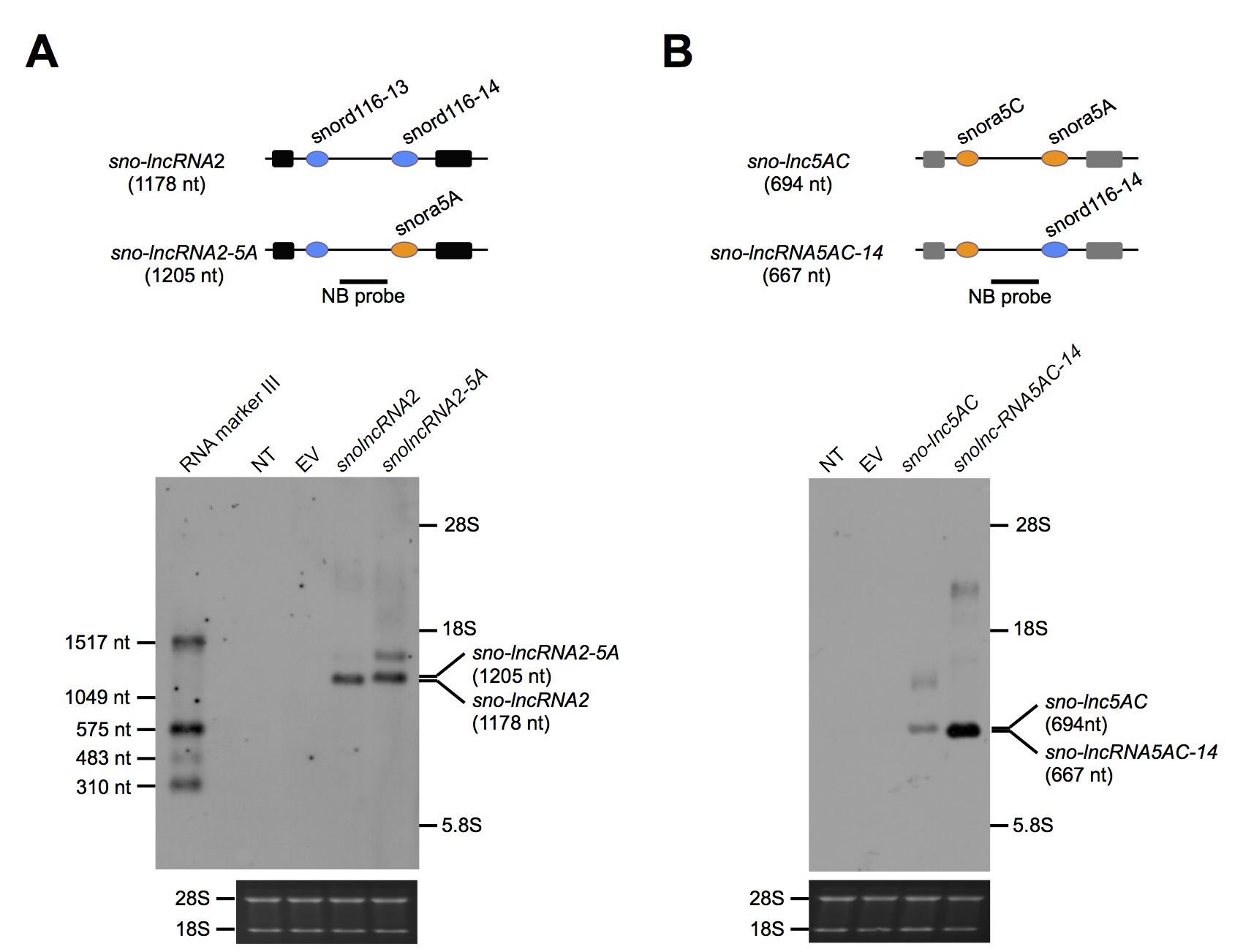

Supplement: Additional file 2 — Northern blots of sno-lncRNAs with native agarose gel. (A) and (B) Northern bolts show that sno-lncRNAs can be recapitulated after replacing the snoRNA end from C/D box snoRNA to H/ACA box snoRNA (A) or vice versa (B). Top, a schematic drawing of wild-type sno-lncRNAs (sno-lncRNA2 and sno-lnc5AC) or modified sno-lncRNAs (sno-lncRNA2-5A and sno-lnc5C-14) in the expression vector. Black/grey boxes, exons; Black bars, NB probes; Blue circles, C/D snoRNAs; Yellow circles, H/ACA snoRNAs; Bottom, Northern blot validation. NT, no transfection; EV, empty vector. RNA marker III was used to indicate RNA sizes. Denatured RNAs were separated on 1% agarose gel. Note that similar RNA separations were obtained by both denatured PAGE gels (Figure 2) and native (shown here) agarose gels. [file 1471-2164-15-287-S2.jpeg]

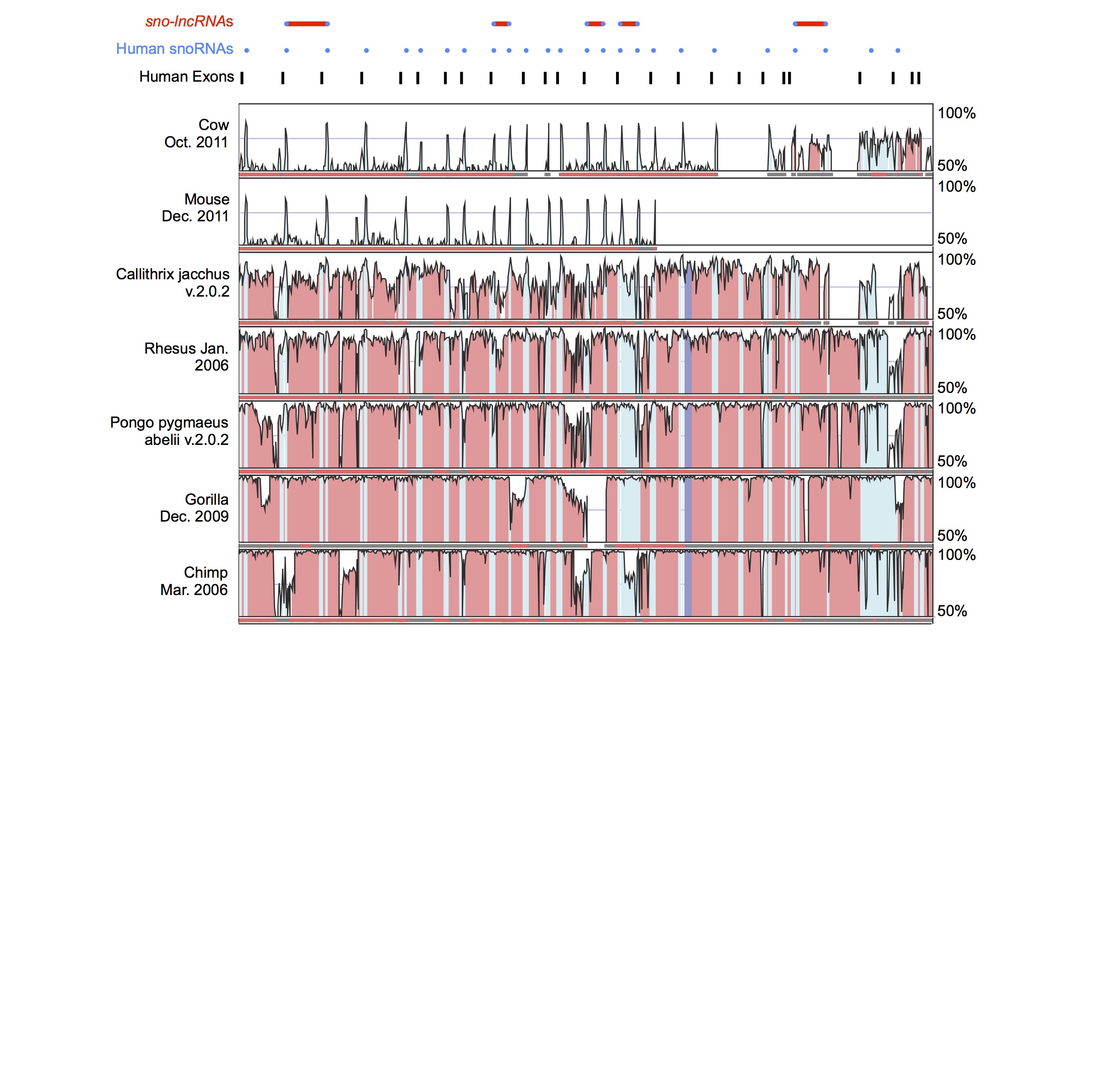

Supplement: Additional file 3 — Sequence conservation analysis of PWS region across species. PWS region snoRNAs (SNORD116 cluster snoRNAs, light blue) exhibit a remarkably higher conservation across species than SNURF-SNRPN exons and introns. Y-axis, species selected for comparing (left panel) and conservation levels (right panel); Red bars, human PWS region sno-lncRNAs; Blue circles, human PWS region snoRNAs (SNORD116 cluster); Black bars, exons of human PWS region sno-lncRNA host gene (SNURF-SNRPN). [file 1471-2164-15-287-S3.jpeg]

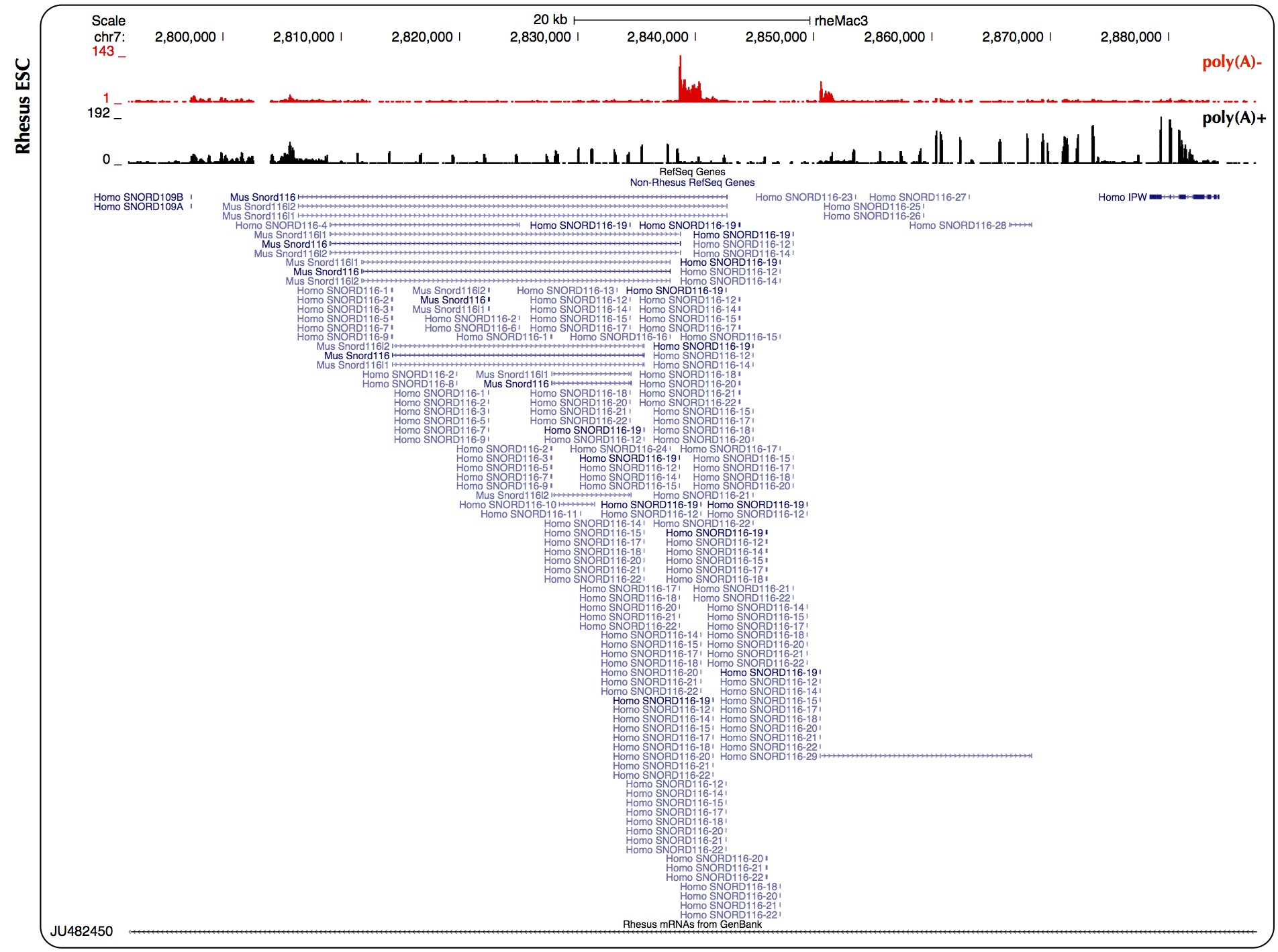

Supplement: Additional file 4 — Expression of PWS region in rhesus. Normalized read densities of poly(A)-/ribo- RNA-seq (red) and poly(A)+ RNA-seq (black) in rhesus ESCs showed two highly expressed sno-lncRNAs in PWS region. Note that there is no RefGene annotation in rhesus, instead, homologues genes from other species are shown. [file 1471-2164-15-287-S4.jpeg]

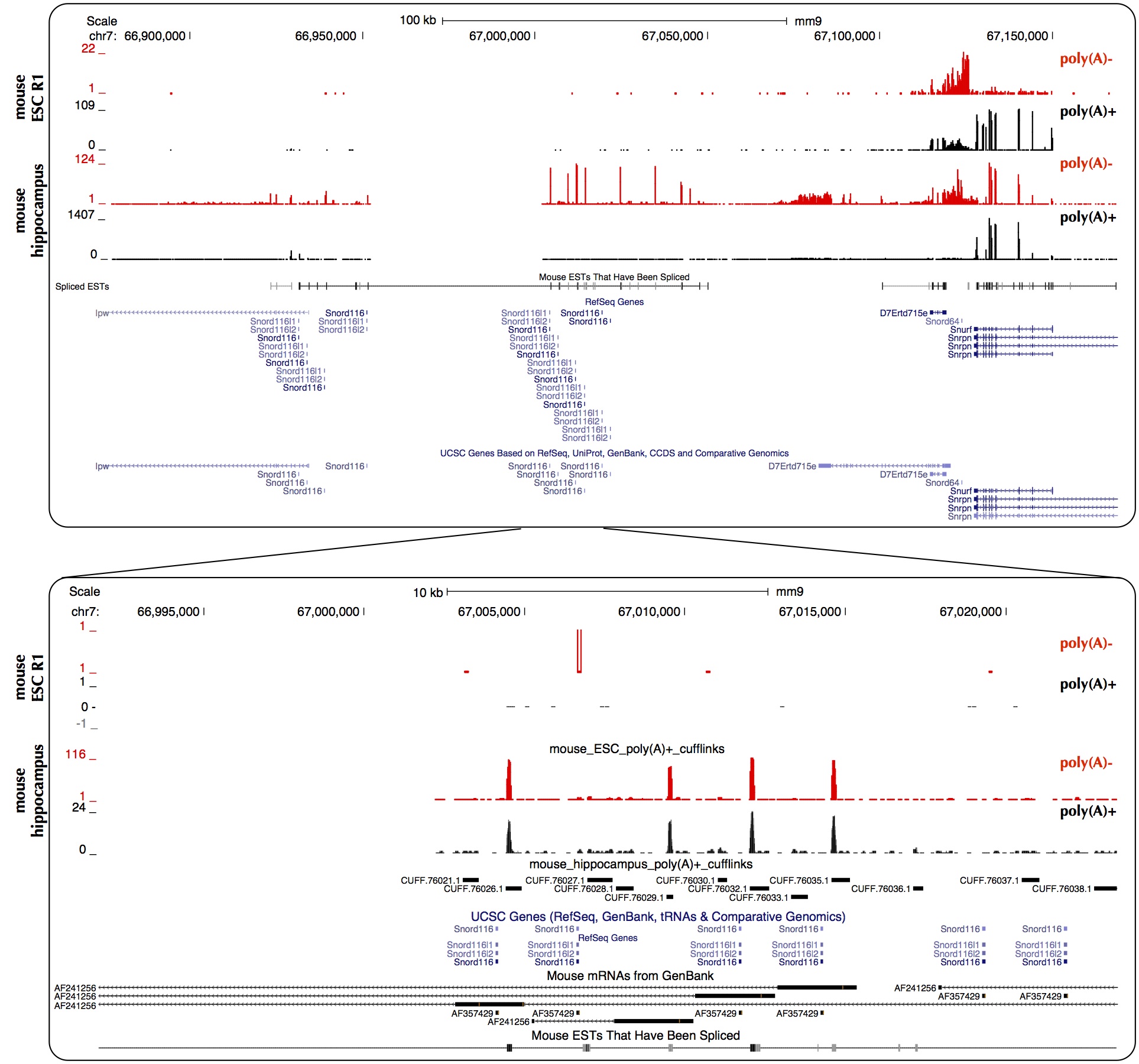

Supplement: Additional file 5 — Expression of PWS region in mouse. Normalized read densities of poly(A)-/ribo- RNA-seq (red) and poly(A)+ RNA-seq (black) of PWS region in mouse ESC R1 and mouse hippocampus showed undetected expression of PWS region sno-lncRNAs. Note that mouse SNORD116 snoRNAs are over 50 kb away from mouse SNURF-SNRPN. These SNORD116 snoRNAs and their adjacent spliced ESTs are not expressed in mESCs, but are expressed in mouse hippocampus. [file 1471-2164-15-287-S5.jpeg]

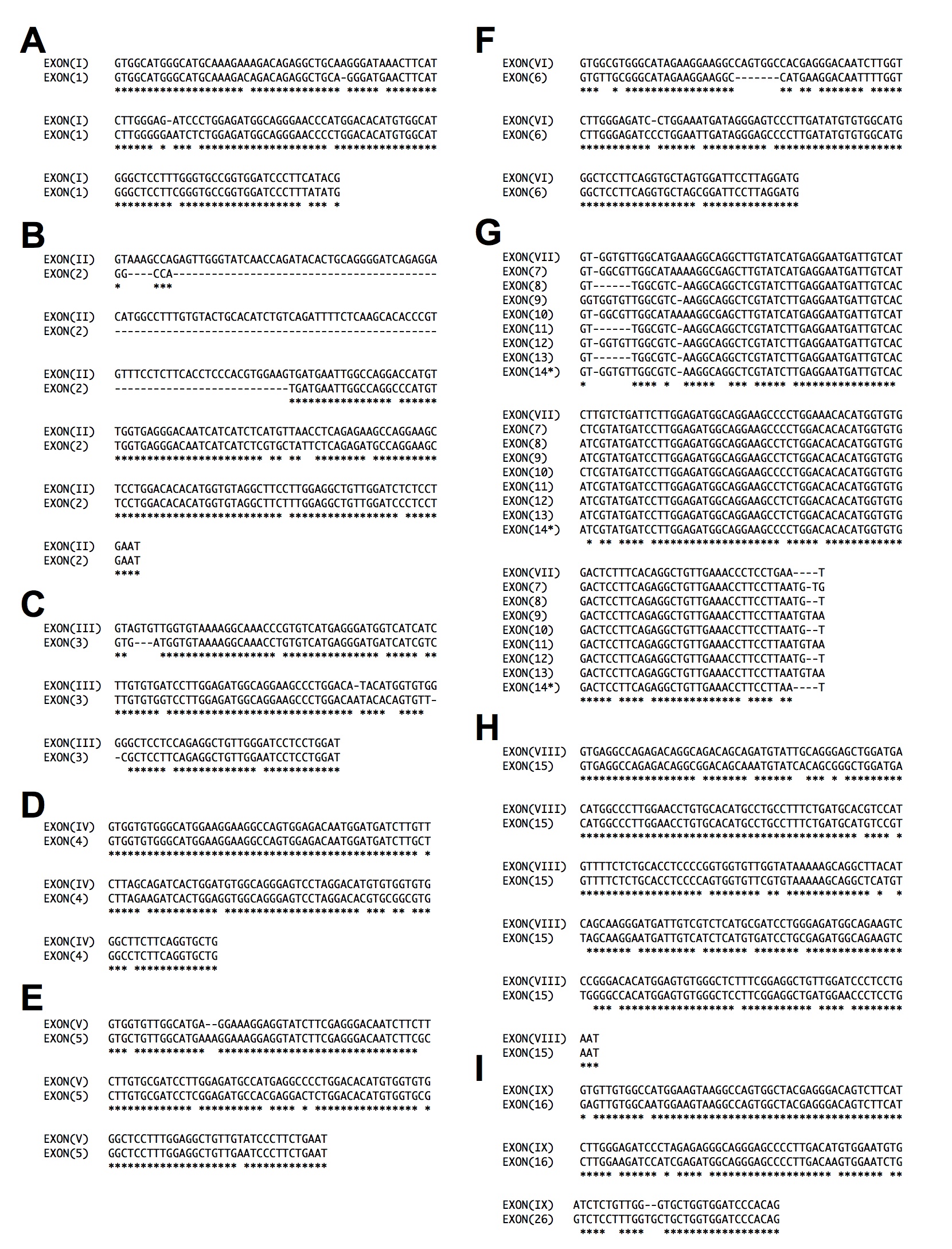

Supplement: Additional file 6 — Pair-wise sequence alignments of SNURF-SNRPN exons between human (black bars of Figure 5A) and rhesus (grey bars of Figure 5A). [file 1471-2164-15-287-S6.jpeg]

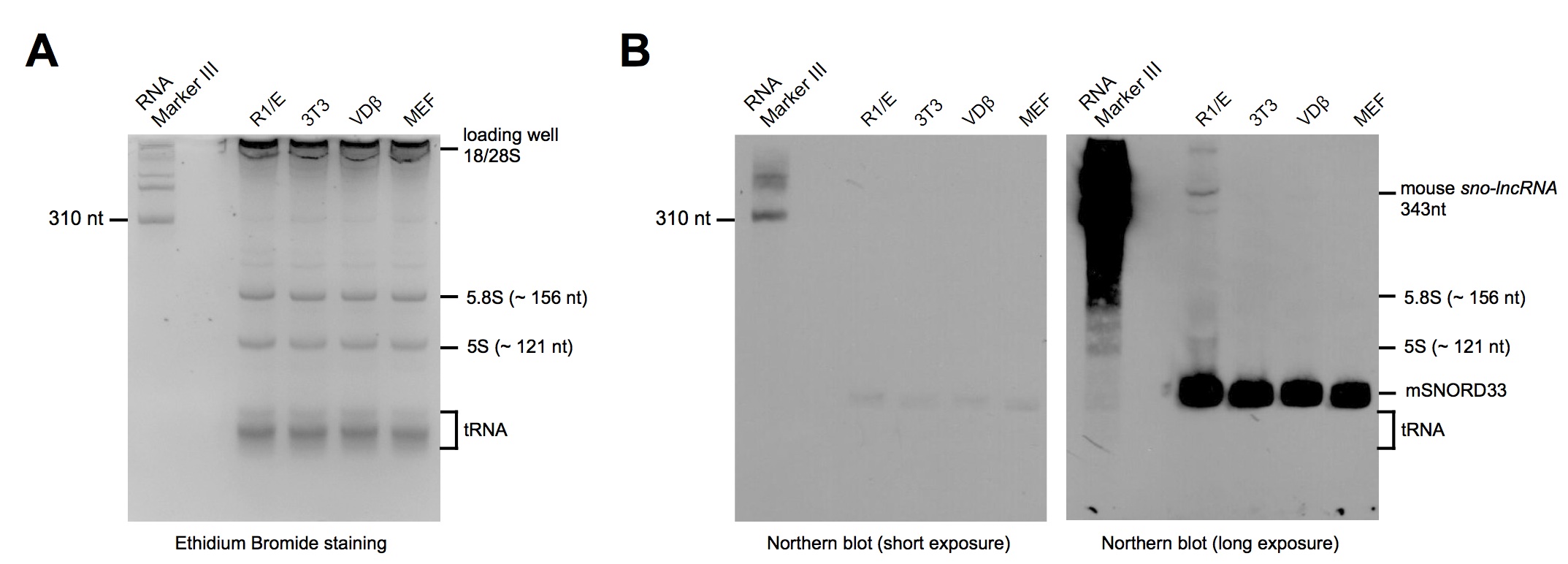

Supplement: Additional file 7 — Northern blot of mouse-specific sno-lncRNA in RPL13A region. Northern blot validation of mouse specific sno-lncRNA from multiple mouse cell lines. Total RNAs from ESC R1, NIH 3T3, VDβ and MEF were denatured and separated on 8% denatured PAGE gel. After separation, the gel was stained with ethidium bromide for rRNA/tRNA visualization (A), and transferred to membrane for Northern blot with probe for SNORD33 (blue bar of Figure 6B) after destaining (B). Positions for 5.8S, 5S rRNA, and tRNAs were indicated with ethidium bromide staining. [file 1471-2164-15-287-S7.jpeg]

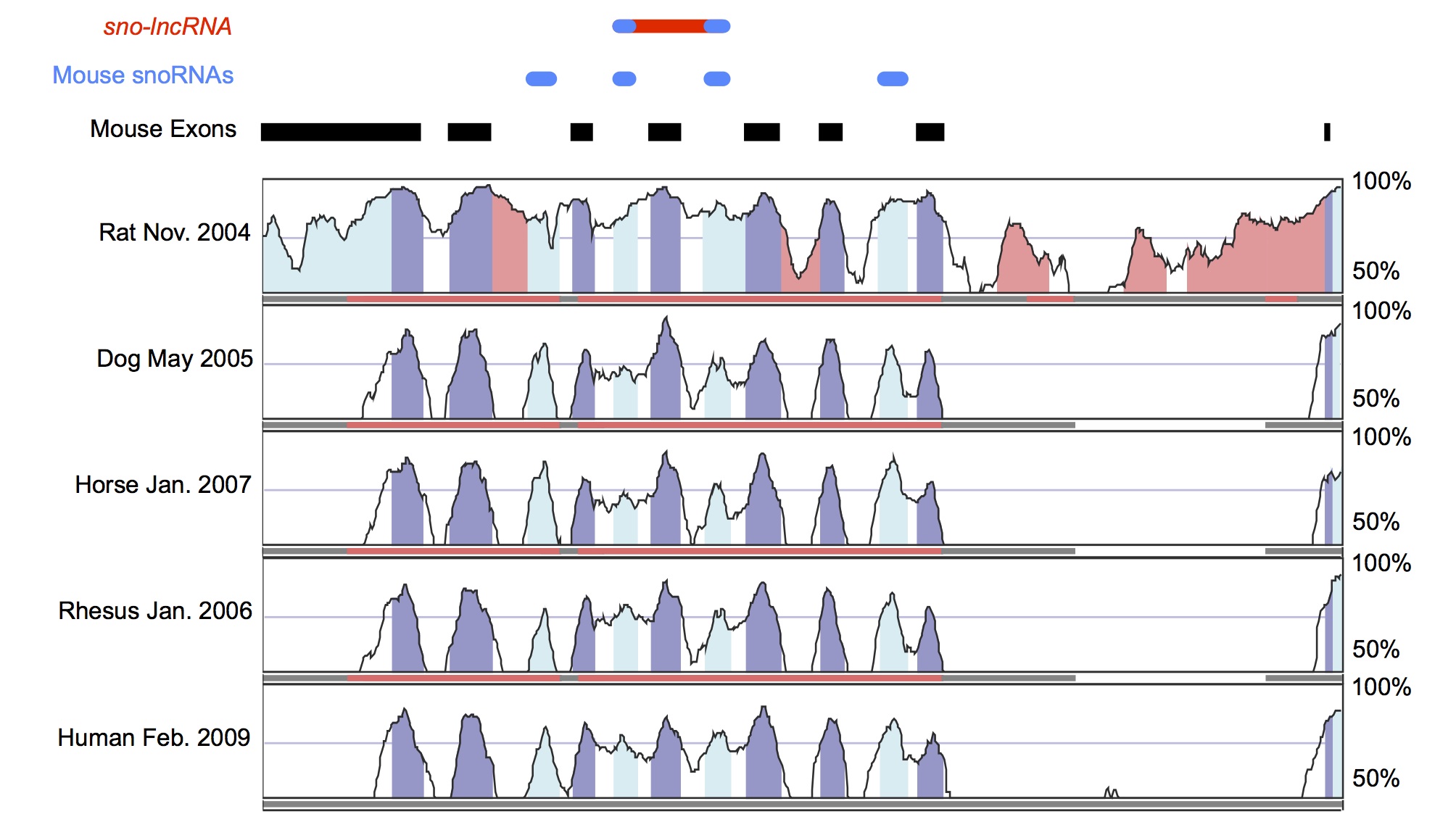

Supplement: Additional file 8 — Sequence conservation analysis of RPL13A region sno-lncRNA. Y-axis, species selected for comparing (left panel) and conservation levels (right panel); Red bars, a non-human sno-lncRNA; Blue circles, mouse snoRNAs (SNORD35, SNORD34, SNORD33 and SNORD32a, from left to right); Black bars, exons of the non-human sno-lncRNA host gene (RPL13A). [file 1471-2164-15-287-S8.jpeg]

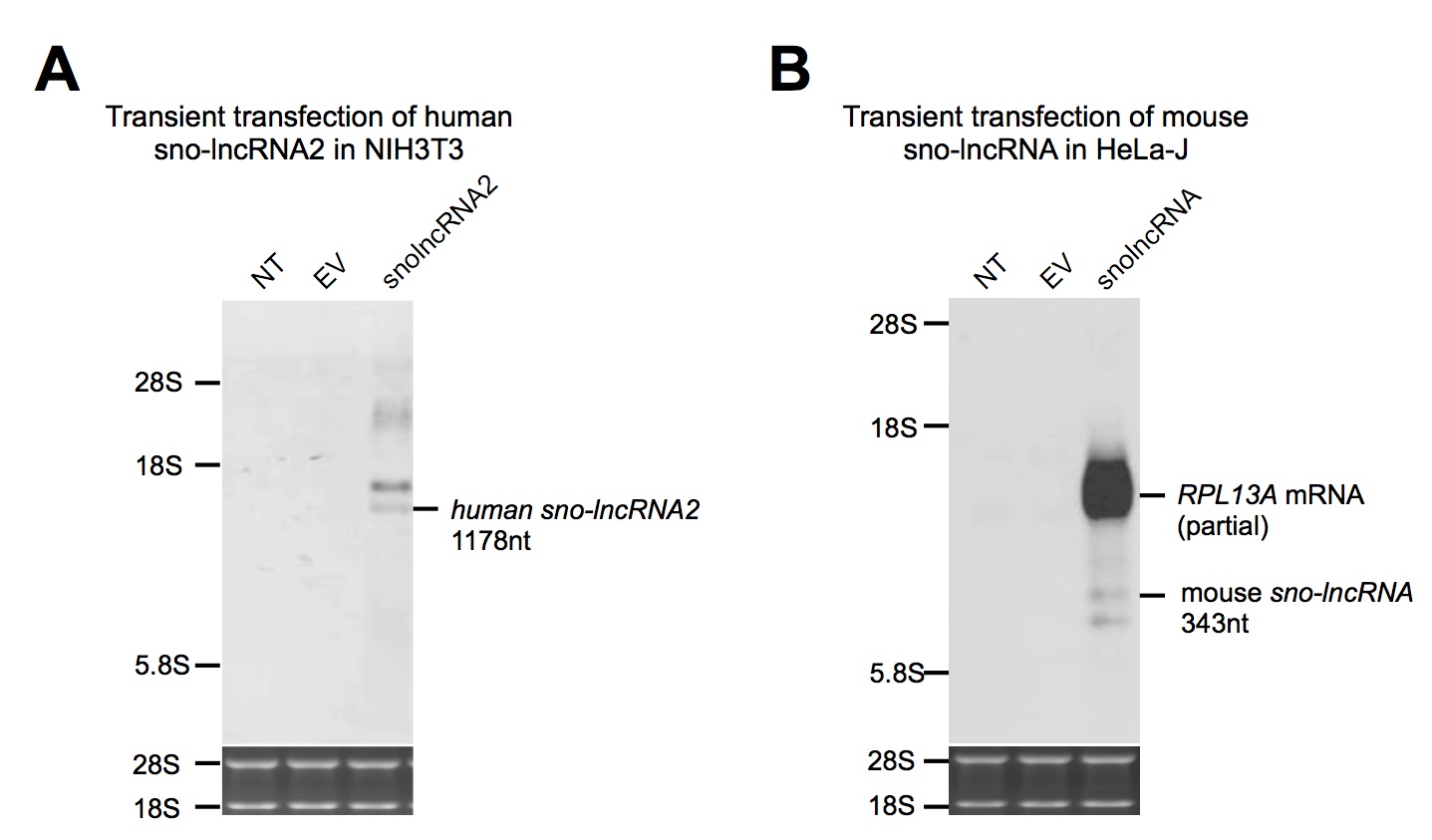

Supplement: Additional file 9 — Transfection of species-specific sno-lncRNA into cell lines derived from different species. (A) Transfection of human sno-lncRNA ito mouse NIH 3T3 cell line generates the human sno-lncRNA as revealed by NB. NT, no transfection; EV, empty vector. (B) Transfection of mouse sno-lncRNA to human HeLa-J cell line generates the mouse sno-lncRNA as revealed by NB. NT, no transfection; EV, empty vector. [file 1471-2164-15-287-S9.jpeg]

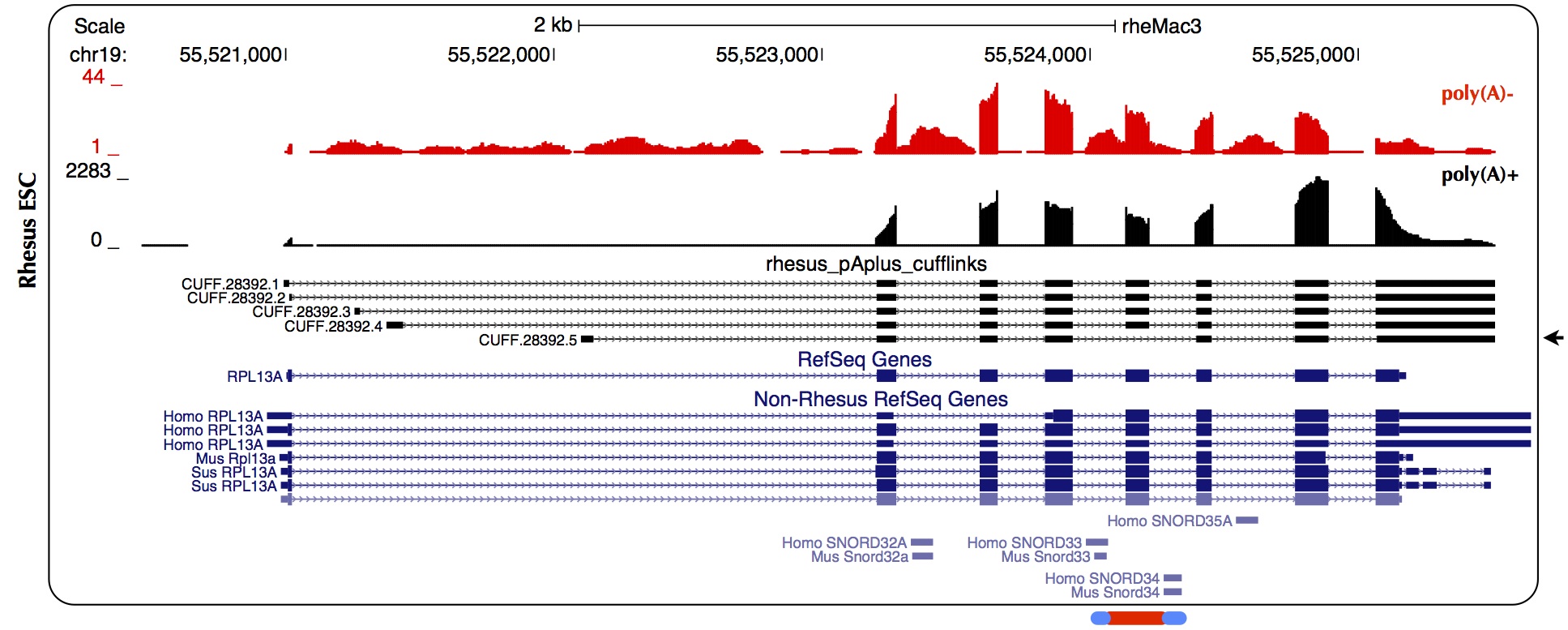

Supplement: Additional file 10 — Species-specific RPL13A region sno-lncRNA is derived from species-specific alternative spliced rpl13a transcripts in rhesus.De novo transcript assembly revealed previously uncharacterized alternative spliced rpl13a transcripts (rhesus_pAplus_cufflinks). One new rhesus rpl13a isoform (indicated by arrow) was identified to splice out a large intron containing SNORD33 and SNORD34. Y-axis, normalized read densities of poly(A)-/ribo- RNA-seq (red) and poly(A)+ RNA-seq (black) of rhesus ESC transcriptomes. [file 1471-2164-15-287-S10.jpeg]
